# Efficient Search Algorithms for Identifying Synergistic Associations in High-Dimensional Datasets

**DOI:** 10.3390/e26110968

**Published:** 2024-11-11

**Authors:** Cillian Hourican, Jie Li, Pashupati P. Mishra, Terho Lehtimäki, Binisha H. Mishra, Mika Kähönen, Olli T. Raitakari, Reijo Laaksonen, Liisa Keltikangas-Järvinen, Markus Juonala, Rick Quax

**Affiliations:** 1 Computational Science Lab, Institute of Informatics, University of Amsterdam, 1012 WP Amsterdam, The Netherlands; jieli198973@gmail.com (J.L.); r.quax@uva.nl (R.Q.); 2 Department of Clinical Chemistry, Faculty of Medicine and Health Technology, Tampere University, 33100 Tampere, Finland; 3 Finnish Cardiovascular Research Center Tampere, Faculty of Medicine and Health Technology, Tampere University, 33100 Tampere, Finland; 4 Department of Clinical Chemistry, Fimlab Laboratories, 33520 Tampere, Finland; 5 Department of Clinical Physiology, Tampere University Hospital, 33520 Tampere, Finland; 6 Research Centre of Applied and Preventive Cardiovascular Medicine, University of Turku, 20014 Turku, Finland; 7 Department of Clinical Physiology and Nuclear Medicine, Turku University Hospital, 33520 Turku, Finland; 8 Centre for Population Health Research, University of Turku and Turku University Hospital, 20520 Turku, Finland; 9 InFLAMES Research Flagship, University of Turku, 20014 Turku, Finland; 10 Zora Biosciences Oy, 02150 Espoo, Finland; 11 Department of Psychology and Logopedics, University of Helsinki, 00100 Helsinki, Finland; 12 Division of Medicine, Turku University Hospital, 20520 Turku, Finland; 13 Department of Medicine, University of Turku, 20014 Turku, Finland; 14 Institute for Advanced Study, 1012 GC Amsterdam, The Netherlands

**Keywords:** synergy, O-information, stochastic search, simulated annealing, particle swarm optimization

## Abstract

In recent years, there has been a notably increased interest in the study of multivariate interactions and emergent higher-order dependencies. This is particularly evident in the context of identifying synergistic sets, which are defined as combinations of elements whose joint interactions result in the emergence of information that is not present in any individual subset of those elements. The scalability of frameworks such as partial information decomposition (PID) and those based on multivariate extensions of mutual information, such as O-information, is limited by combinational explosion in the number of sets that must be assessed. In order to address these challenges, we propose a novel approach that utilises stochastic search strategies in order to identify synergistic triplets within datasets. Furthermore, the methodology is extensible to larger sets and various synergy measures. By employing stochastic search, our approach circumvents the constraints of exhaustive enumeration, offering a scalable and efficient means to uncover intricate dependencies. The flexibility of our method is illustrated through its application to two epidemiological datasets: The Young Finns Study and the UK Biobank Nuclear Magnetic Resonance (NMR) data. Additionally, we present a heuristic for reducing the number of synergistic sets to analyse in large datasets by excluding sets with overlapping information. We also illustrate the risks of performing a feature selection before assessing synergistic information in the system.

## 1. Introduction

Synergistic associations are complementary to pairwise associations. While pairwise associations are seen as the information gained from one variable by studying the state of another, a synergistic association is the information provided by a group of variables together that is not found between any pair of variables. Both types of association are necessary ingredients to analyse complex systems. While the analysis of pairwise associations is commonplace, synergistic associations have recently received more attention, and have been found in several different domains, such as neuroscience [1,2,3], cardiovascular disease [4], multimorbidity [5], ageing [6] and psychometrics [7], among others.

The problem is that evaluating all synergistic associations in a dataset requires evaluating all subsets of variables and computing a synergy score. This exponential growth limits the applicability of these approaches on large-scale datasets. For example, a dataset of 400 variables has 10,586,800 sets of triplets and 1,050,739,900 sets of quadruplets. This means that searching for higher-order associations beyond three variables has, until now, remained out of reach for many larger datasets. Indeed, many related works confine their search to a smaller set of variables in order to make their study feasible. We argue that this can sometimes be inappropriate, as it may result in synergistic associations remaining undiscovered.

We propose to identify the strongest synergistic associations by making use of global optimization algorithms. We employ two stochastic search strategies, as well as a direct network-based approach. The main idea is that the brute-force method to identify the strongest synergistic associations of order *n* (i.e., among *n* variables) would require a combinatorial number of calculations. Instead, the search algorithms will be guided by lower-order features (i.e., statistical quantities computed on sets of variables of size less than *n*), which is much less time-consuming. The hypothesis is that the search algorithms are still capable of predicting the top synergistic associations with reasonable accuracy. Note that focusing on only the top synergistic associations simplifies the search task compared to brute force because a total ordering is not sought, only a partial order of a small number of associations.

The goal of understanding synergistic relationships between groups of variables has led to the formulation of algorithms for computing (the lower bound of) the amount of synergistic information in a given set, while also considering discretization, bias, and sample-size effects. However, there is currently no universally accepted metric in the informatics community for computing synergistic associations. Multiple heuristics exist for computing synergy, such as those based on partial information decomposition (PID) [8,9]. Alternative approaches are also computationally expensive, requiring various optimizations for computing synergistic random variables [10] or finding geodesics between sub-manifolds [11]. Even for more lightweight approaches, such as the O-information [12], the time complexity for computing sets of size *p* on a dataset with *k* variables is $O(k^{p})$. Computing all possible sets has a complexity of $O(2^{k})$. This limits the applicability of these approaches on large-scale datasets. Our approaches are evaluated on two popular approaches: bias-corrected O-information, and a PID-based approach, the latter of which includes an optimization step. We stress that our approach is general, and is not limited to any particular synergy measure.

### 1.1. Search Algorithms

There exist a wide range of optimization algorithms for finding local minima in data. In this paper, we explore the use of Simulated Annealing (SA) and Particle Swarm Optimization (PSO) to find synergistic associations. In addition to stochastic optimization techniques, we also investigate whether a more direct network-based approach could be used to find synergistic associations. For this, we constructed a network of order n−1 (e.g., pairwise) metrics (e.g., conditional entropy or mutual information), which could be used to identify order *n* (e.g., triplet) synergistic associations. The predictive n−1 feature is found through model selection, where a regression model is trained on a selection of n−1 features to predict synergistic information. We then define a clique-based heuristic algorithm that searches for triangles in a network which are likely to contain synergistic information. This approach allows us to quickly identify a set of potentially synergistic associations, although the strongest synergistic sets are not necessarily found.

### 1.2. The Echo Problem

We discover a complication that arises when identifying synergistic associations in data, which we refer to as the echo problem. That is, whenever a strong pairwise association is connected to a synergistic n-plet, then an additional n-plet will necessarily be found, since the endpoints of the pairwise association are (almost) interchangeable. Since any given dataset consists of both pairwise associations and synergistic associations, this can potentially lead to an explosion of identified n-plets, even if the underlying data generating mechanism would contain only few synergistic n-plets. We consider such synergistic n-plets, which are only present due to connecting pairwise links, as spurious. In the literature, this appears to be a so-far underappreciated aspect of inferring higher-order interactions and information-theoretic hypergraphs [7]. We show that echoes significantly impact the global network structure. They lead to different central node sets, varied path length distributions, and changes in clustering. This is highly relevant for downstream inference. Therefore, we propose an additional regularization step that is incorporated into the global optimization.

The purpose of this paper is two-fold: first, to efficiently identify the most synergistic associations in the data, and second, to filter out synergistic sets with overlapping associations. We assess the performance of our search strategies by comparing their outcomes to the brute force results. Specifically, we compare the most synergistic triplets found by the search strategies to the distribution of all triplet scores in the data. We also compare the running times and number of calls to the synergy metric. We test our approaches on two datasets: the Cardiovascular Risk in the Young Finns Study (YFS) [13], and NMR data from the UK Biobank [14].

## 2. Background

### 2.1. Measuring Synergy

We selected two metrics to implement the search algorithms: the bivariate PID measure of Makkeh et al. [15], which we estimate using the idtxl package [16], and the O-information metric [12]. This PID-metric solves a convex optimization problem to decompose the mutual information MI(X:Y,Z) into synergistic, redundant, and unique components. The synergistic part is always non-negative. On the other hand, the O-information metric is a direct calculation that aims to quantify the balance (arithmetic difference) between synergy and redundancy. Therefore, its value can be negative (synergy-dominated) or positive (redundancy-dominated). O-information has the benefit of being instantaneous to compute (no optimization needed), but it is conservative, i.e., some interactions may be at least partly synergistic, but the O-information measure fails to detect them [12] in cases where redundant information is also present. The converse is not possible: if O-information infers a synergistic relationship, then there must be significant synergy, i.e., there must be more synergy than redundancy. This metric, and its variants, have recently seen a surge of interest, especially in neuroscience [1,2,17,18,19,20,21]. It is important to note that, in this article, we are only concerned with assessing whether search algorithms are a good approximation of a brute force approach in ranking synergistic associations. In particular, we do not compare the synergy values or rankings between the methods, since they are not expected to give the same results.

### 2.2. Simulated Annealing and Particle Swarm Optimization

Simulated Annealing (SA) is a stochastic optimization algorithm designed to converge gradually toward a global minimum. In this context, the global minimum represents the most synergistic *n*-plet (subset) within a dataset for a specified set size *n*. However, due to the tendency of SA to become trapped in local minima, multiple runs are often necessary. To address this limitation, PSO offers an alternative approach. In PSO, multiple “particles” simultaneously explore the search space, each sharing information about their individual successes with the group. This collaborative mechanism makes PSO highly parallelizable and scalable, and less likely to converge prematurely at local optima. By maintaining multiple particles in the search space, PSO reduces the likelihood of being confined to local minima, thus enhancing its robustness for finding global minima in complex, multimodal landscapes. SA was applied in [22] to search for synergistic information using O-information. Our extension builds on this methodology, directing the search toward synergistic sets by incorporating pairwise metrics into the decision-making process for proposing new sets at each step of the algorithm. To our knowledge, no such search strategy has been applied to other synergy metrics.

SA and PSO are metaheuristic algorithms commonly used for global optimization tasks. SA operates by initially assigning a high “temperature” that gradually “cools”, allowing for a broad exploration of the solution space initially, followed by a narrowing focus on the promising areas. At the beginning of the search, SA probabilistically accepts poorer solutions to help escape local optima, with this acceptance probability diminishing as the temperature decreases. In contrast, PSO initializes a population of candidate solutions, or “particles”, that move within the search space according to both their own best-known positions and the swarm’s best-known position [23]. Each particle adjusts its trajectory based on these two factors, allowing the swarm to effectively navigate the solution space in search of an optimal solution. Although PSO is primarily used in continuous spaces, variants have been developed for applications in discrete spaces or set-based frameworks to address combinatorial optimization problems [24,25,26].

## 3. Methods

We use a regression model trained specifically on pairwise metrics to predict synergy scores in triplets. This procedure can easily be extended to higher orders. The objective was to assess whether combinations of pairwise metrics are predictive of the presence of synergistic triplets and, if so, to transform them into pairwise-based metrics for biasing the search direction of the global search algorithms. Based on the identified pairwise metrics, we then developed three search strategies to find synergistic associations: two probabilistic search strategies (PSO and SA), and one deterministic clique-based algorithm.

### 3.1. Dataset Preparation

We test the effectiveness of the search algorithms on two biomedical data sets with different focus areas: the NMR data from the UK Biobank [14], and the Cardiovascular Risk data in the Young Finns Study (YFS) [13].

- The **UK Biobank** is a large-scale biomedical database and research resource containing in-depth genetic and health information from half a million UK participants. Nuclear Magnetic Resonance (NMR) spectroscopy data in the UK Biobank refer to metabolic profiling of blood samples. The NMR spectroscopy provides detailed information on lipoproteins, metabolites, and other molecules in the blood.
- **YFS** is an epidemiological cohort study focusing on the risk factors for cardiovascular diseases starting from childhood. The study aims to understand how childhood lifestyle, biological factors, and social environment influence cardiovascular health in adulthood. It contains clinical assessments, psychological evaluations, lifestyle questionnaires, and biological samples, tracking participants from childhood to adulthood.

For computing synergy values based on O-information, we used the continuous variables from the UKB NMR dataset, and discretized them using quantile binning using the Sturges rule. Discretization was performed so that meaningful entropy-based features could be computed, and quantile binning was used to reduce any biases arising from the number of bins used.

The UK Biobank NMR dataset consists of 336 participants, with detailed metabolomic data obtained through Nuclear Magnetic Resonance (NMR) spectroscopy. Variables with 20% missing entries were removed, leaving 1426 variables. For variables with less than 20% missing data, missing values were imputed using a multiple imputation approach with a K-Nearest Neighbours Regressor, using the IterativeImputer from scikit-learn. Imputation was performed iteratively, with a maximum of 1000 iterations and a convergence threshold of 0.01. If the average change between successive imputations was below the threshold, convergence was achieved; otherwise, the imputation process continued for up to 5 iterations. For computing synergy values based on the PID framework, we used four states per variable, because the computational cost scales with the number of states per variable.

In the YFS study, data from 1684 participants were used, and continuous variables were discretized in the same manner. No imputation was performed. When searching for O-information synergy, 377 variables were used from the 2007 wave. Existing discrete variables, such as gender and depression scores, were also included, so that the search algorithms could be assessed on data with non-uniform entropy distributions. When searching for PID-based synergy, 684 variables were used from the 2001, 2007, and 2011 waves, and all were discretized into five states. However, only variables that could be discretized into exactly five states were used.

### 3.2. Using Lower-Order Information-Theoretic Metrics to Predict Synergistic Associations

We computed several variants of pairwise metrics with aggregation functions such as minimum, maximum and average, as well as all triplet synergies (brute force) in both the UKB NMR and YFS datasets. This resulted in a multi-regression problem of predicting a triplet’s synergy based on metrics such as the minimum pairwise mutual information, sum of joint entropies, and spread of mutual information scores. We used the Light Gradient Boosting Machine (LightGBM), and performed a grid search of hyperparameters using 5-fold cross-validation minimization for the mean squared error.

Recursive feature elimination with cross-validation (RFECV) was used to perform a feature selection on our gradient boosting model. This process systematically discards the least important features, splitting the data into five folds to iteratively train the model and evaluate its performance using RMSE. Following RFECV, we evaluated the performances of each combination of the significant features identified by RFECV using a grid search to minimize RMSE. After feature selection, the *train_test_split* method from the scikit-learn library [27] was used to create a single split of 70% training data and 30% test data for evaluating all possible feature combinations. The model using the best found hyperparameters for each combination was then fitted to the training data. By evaluating each feature combination, we could identify the most effective feature subsets to use in our search strategies.

### 3.3. Simulated Annealing for Synergistic Associations

SA is a probabilistic algorithm for approximating the global optimum of a function by gradually cooling the search process. The algorithm begins by initializing a particle at a random position, $X^{0}=\left\{x_{j},x_{k},\cdots ,x_{m}\right\}$, within the search space of all sets of the desired cardinality. For example, when searching for synergistic triplets, each set’s cardinality is three, with each $x_{j}$ representing a unique variable in the dataset. This initial position is evaluated using a synergy scoring function, $\Omega (X^{0})$.

In each iteration, a new candidate position $\hat{X^{t}}$ is proposed by modifying one or more variables in $X^{t}$, with the number of changes following a Gaussian distribution. The spread of this distribution dictates the number of variables to alter. The newly proposed set, $\hat{X^{t}}$, is then evaluated. If $\Omega \left(\hat{X^{t}}\right)<\Omega \left(X^{t}\right)$, indicating a more synergistic set, $\hat{X^{t}}$ replaces $X^{t}$ as the current set. If the candidate set is less optimal, it may still be accepted with a probability $\exp\left(\frac{\Omega \left(\hat{X^{t}}\right)-\Omega \left(X^{t}\right)}{T}\right)$, where *T* is the algorithm’s temperature, which decreases over time. This cooling schedule lowers the acceptance rate of suboptimal sets as *T* approaches zero, allowing for broad exploration early on (when *T* is high) to avoid local minima, and refining toward an optimal solution as *T* cools.

Originally applied in [22] to identify synergistic triplets using the O-information metric, our SA algorithm extends this method by incorporating non-uniform distributions for selecting variables in new sets. Each SA run begins with a random variable set, which is evaluated to determine an initial synergy score. The algorithm then explores the search space iteratively by proposing new sets, using the Metropolis acceptance criterion based on synergy difference and current temperature.

At each iteration, the number of components to modify is determined based on the current temperature, following a geometric distribution. The probability parameter *p* for this distribution is defined by ${\left(\frac{\mathrm{current}\;\mathrm{temperature}}{\mathrm{initial}\;\mathrm{temperature}}\right)}^{0.1}$, ensuring that *p* remains within (0,1]. The number of variables to change is then sampled based on this probability.

Once the number of variables to swap is set, the algorithm identifies which specific variables to replace. If one variable is to be replaced, it is selected probabilistically, based on the weights derived from the inverse mutual information between each variable in the current set and the candidate variables. If two variables are to be replaced, the pairwise inverse mutual information is similarly used to determine the selection probabilities. Specifically, the inverse mutual information values between each pair of current and candidate variables are calculated, normalized into a probability distribution, and used to sample the variable(s) for replacement.

For example, if two variables are to be replaced based on the inverse mutual information metric, the algorithm calculates the inverse mutual information between each variable in the current set and all candidate variables. Variables that exhibit lower mutual information (higher inverse mutual information) are more likely to be replaced, thereby enhancing synergy by favouring replacements that reduce redundancy. This selection method guides the search toward more synergistic sets by reducing the information overlap.

The adjustments in the replacement strategy and probability distribution in our SA approach are informed by insights from the regression model analyses, which identified key pairwise metrics that significantly enhance the optimization process.

### 3.4. Particle Swarm Optimization for Synergistic Associations

The PSO algorithm uses a collection of *P* particles to explore the search space for synergistic associations. Each particle starts at a random position, where a position represents a specific set of variables used to calculate synergy. Particles track their best positions and scores (personal bests) while also accessing the global best position and score identified by any particle in the swarm. The swarm iteratively updates particle positions based on their current velocity ($V_{i}$), their historical best-known positions ($P_{{\mathrm{local}}_{i}}$), and the swarm-wide best-known position ($P_{\mathrm{global}}$).

At each iteration, particle *i* considers a set of three variables. This set, denoted $X_{i}^{t}$, is scored based on the synergy of the variables it contains. If the current score at $X_{i}^{t}$ is better than the particle’s personal best, it updates its record. If this score also exceeds the global best, the global best position is updated for the swarm,

$$\mathrm{If}\;\Omega \left(X_{i}^{t}\right)>\Omega \left(X_{{\mathrm{local}}_{i}}\right),\;\mathrm{then}\;X_{{\mathrm{local}}_{i}}=X_{i}^{t}$$

$$\mathrm{If}\;\Omega \left(X_{{\mathrm{local}}_{i}}\right)>\Omega \left(X_{\mathrm{global}}\right),\;\mathrm{then}\;X_{\mathrm{global}}=X_{{\mathrm{local}}_{i}}$$

Particles move probabilistically at each step, similarly to the SA approach. A normal distribution sample determines how many components to change in the current position. The further the sampled value is from the mean, the more elements are replaced in $X_{i}^{t}$. When within one standard deviation, a single variable in $X_{i}^{t}$ is replaced. This replacement is selected based on a blended probability distribution that considers mutual information with other candidate variables, and nudges particles toward both the global best and their local best positions. This blended approach encourages particles to explore promising regions influenced by both personal success and swarm success.

#### 3.4.1. Probability Distribution Update

The initial probability distribution $P=[p_{1},p_{2},\ldots ,p_{n}]$ is derived from pairwise metrics such as mutual information. This distribution is then adjusted to incorporate information from the current local and global bests, forming an updated distribution $P^{\prime }$. Parameters α and β modulate the influence of the local and global bests, respectively. Blended weights are calculated by combining the original distribution with these “nudge” distributions,

$$\mathrm{blended}\text{\_}\mathrm{weights}=\left(1-\mathrm{nudge}\text{\_}\mathrm{factor}\right)\times P+\alpha \cdot \Delta_{\mathrm{local}}+\beta \cdot \Delta_{\mathrm{global}}$$

The updated probability distribution $P^{\prime }$ is then normalized,

$$P^{\prime }=\frac{\mathrm{blended}\text{\_}\mathrm{weights}}{\sum_{i=1}^{n}{\mathrm{blended}\text{\_}\mathrm{weights}}_{i}}$$

This probability update was developed from insights in our regression analysis, identifying the pairwise metrics critical for effective optimization. By integrating these metrics, PSO’s probability distributions and adaptive nudging parameters are refined, allowing for particles to be directed more effectively towards promising search areas, and enhancing both the efficiency and accuracy in the synergy search.

#### 3.4.2. Particle Movement Strategies

Various movement strategies were implemented using different pairwise metrics to compute the candidate probabilities before applying the nudges. These strategies include uniform probabilities, mutual information between the removed node and candidate nodes, inverse mutual information between the removed node and candidate nodes, mean (inverse) mutual information between the remaining nodes and candidate nodes, mean mutual information between remaining nodes and candidate nodes, and a combined strategy using the product of the entropy and the mutual information. Blended probabilities are computed as described in Section 3.4.1, which balance the current movement direction (influenced by mutual information) with a tendency to explore new areas based on both local and global best positions.

Four nudging strategies were also explored to balance exploration and exploitation. No nudging acted as a baseline, where particles move independently of the local and global bests. Constant nudging provided a fixed nudge factor directing particles towards local and global bests. With exponential decay nudging, the nudge strengths decrease over time, according to

$$\mathrm{LocalNudge}=\mathrm{GlobalNudge}=\mathrm{NudgeFactor}\cdot e^{-\frac{t}{\max\text{\_}\mathrm{steps}}}$$

While in an adaptive nudging strategy, the nudge strengths adjust dynamically, guiding particles towards regions with high synergy based on the current performance and the proximity to the best scores. Nudge factors α and β are dynamically adjusted based on particle performance and proximity to the best scores, using sigmoid functions,

$$\mathrm{Local}\;\mathrm{Nudge}=\sigma \left(\frac{t}{\max\text{\_}\mathrm{steps}}+\alpha \cdot ∥{\cos t}_{i}-\mathrm{global}\text{\_}\mathrm{best}\text{\_}\mathrm{score}∥\right)$$

$$\mathrm{Global}\;\mathrm{Nudge}=\sigma \left(\frac{t}{\max\text{\_}\mathrm{steps}}+\beta \cdot ∥{\cos t}_{i}-{\mathrm{particle}\text{\_}\mathrm{best}\text{\_}\mathrm{score}}_{i}∥\right)$$

where $\sigma \left(x\right)=\frac{1}{1+e^{-k\cdot x}}$ is the sigmoid function, and α, β, and *k* are scaling factors.

### 3.5. A Clique-Based Heuristic to Identify Synergistic Interactions

The third and final approach we have developed is a clique-based heuristic algorithm. The aim of this approach is to more directly identify the synergistic interactions without involving a stochastic search strategies or brute force calculations. In this iterative approach, we construct a pairwise network by adding edges based on an aggregated pairwise information-theoretic measure. Suppose it turns out that a high synergy is associated with low pairwise conditional entropies. Then, it stands to reason that each pair of variables in a synergistic triplet will have a low conditional entropy. Therefore, we construct the network edge-by-edge, in descending order of the sorted values for the correlated pairwise metric. For example, when computing triplets, we continue until a specified number of three-cliques (i.e., triangles) are present in the network. These cliques are then considered as potential synergistic n-plets (sets). It then keeps track of cliques formed during the process, and continues until a maximum of 1000 cliques are identified, which is sufficient for our purposes. The result is an ordered list of cliques extracted from the graph, each representing a potentially synergistic set. A discussion of the algorithms computational complexity is given in Appendix Clique Algorithm Complexity.

### 3.6. Reducing the Number of Synergistic Associations to Analyse

We must access the synergy for each set *X* of *p* nodes, where Ω(X)<0. Our objectives are twofold: we seek to confirm that each constituent node in the set contributes significantly to the synergistic information, while simultaneously ensuring that this information is non-redundant with respect to other established sets.

To determine the statistical significance of a synergistic set, a permutation test or a bootstrapping procedure is often employed [7]. When employing the bootstrapping method, the nodes that yield a confidence interval excluding zero for the O-information value, combined with a *p*-value falling below a predetermined significance threshold, are categorized as significantly synergistic. However, when a large number of echoes are present in the data, resulting in an inflated number of synergistic triplets, a large (and often unfeasible) *p*-value adjustment may be needed to correct for the (potential) false discovery rates when making multiple comparisons. By first removing the echoes, it becomes feasible to test the statistical significance of the synergistic sets.

Once the synergy scores have been computed for all sets of order *p*, we sort them by decreasing amounts of synergy, and we initialize an empty set for the accepted synergistic sets. We then enter a loop that continues until there are no remaining sets to process. In each iteration, the most synergistic set is selected. For each set in the sorted data frame, if the set has all but one element overlapping with the most synergistic set, the synergy of the merged set (union of the two sets) is computed. If the merged set’s synergy is less than that of the overlapping set, the overlapping set is marked for rejection. At the end of each iteration, the most synergistic set is added to the accepted sets if it has not been rejected, and all sets marked for rejection are removed from the data frame. This process repeats, updating the data frame by removing accepted and rejected sets, until no more sets remain to be processed. The final result is a list of accepted synergistic sets.

We argue against removing variables prior to computation merely because they are highly correlated. Although highly correlated variables often exhibit similar pairwise associations with other variables, this pattern does not necessarily extend to higher-order associations. Later, we present examples of strongly correlated variables with distinct synergies, demonstrating how removing one variable can result in the omission of important synergies from the analysis.

### 3.7. Improvement Factor for Qualitative Biological Insights

In addition to identifying the top triplets with the lowest bias-corrected O-information, we use the improvement factor to assess the biological relevance of certain biomarker interactions. The improvement factor is calculated as the ratio of the joint mutual information (MI) of two variables about a third variable to the sum of their individual pairwise MI values. When the improvement factor exceeds 1, it suggests that the combination of two variables provides additional, unique information about the third, beyond what each variable contributes independently. Higher improvement factors, therefore, indicate stronger synergies, where interactions between variables reveal insights into complex, higher-order relationships not captured by the pairwise associations alone.

In this study, the top ten triplets with the largest improvement factors were analysed alongside the top ten triplets with the lowest O-information to identify robust higher-order interactions relevant to lipid metabolism. By examining triplets with high improvement factors, we gained insight into interactions that might be relevant to lipid homeostasis and metabolic processes, beyond what the pairwise correlations alone reveal.

## 4. Results

### 4.1. The Prevalence of Synergy in Data

**Figure 1:**
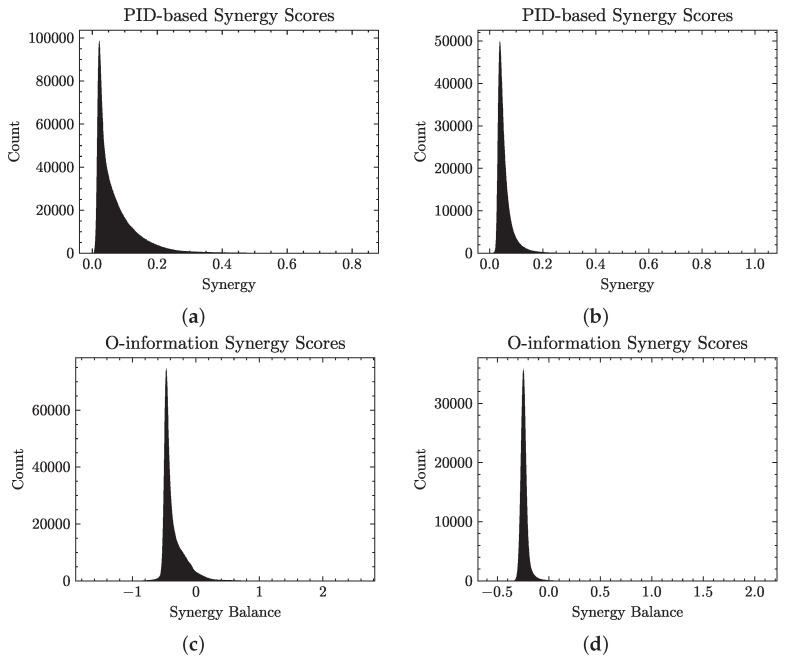
Distribution of synergy scores across the YFS dataset and the NMR variables in the UKB data, using two different synergistic metrics and different data binning. The PID-based approach in (**a**,**b**) shows a notable skew towards lower values. The data exhibits a heavy right tail, indicating that higher synergy scores are less common. Similarly, in (**c**,**d**), the majority of triplets have a very low synergy score (near zero). In the UKB NMR data, only 0.4% have a strongly synergistic score below −0.7, while the minimum synergy score in the YFS data is −0.56. (**a**) UKB NMR PID-based synergy scores using data discretized into five states via quantile binning. (**b**) YFS PID-based synergy scores using data discretized into four states via quantile binning. (**c**) UKB NMR O-information synergy-redundancy balance scores using data discretized into 12 states via quantile binning. (**d**) YFS NMR O-information synergy-redundancy balance scores using data discretized into 12 states via quantile binning.

Figure 1 illustrates the distribution of PID-based synergy and O-information scores in the UKB NMR data. In Figure 1a, the distribution shows that the majority of synergy scores are concentrated near the lower end of the scale, with a rapid decline in the frequency as the score increases. This results in a highly skewed distribution with a long right tail, signifying that high synergy scores are relatively rare within the dataset; 5.8% of triplets had a synergy score above 0.2. This skewed distribution pattern suggests that most interactions within the dataset exhibit a low synergy, while a few interactions achieve a significantly higher synergy, potentially representing unique cases of high collaborative effects. The O-information scores in Figure 1c tell a similar story, where the majority of triplets have negative scores, indicating possible synergy; however, only a small subset of these are strongly synergistic. The mean score is −0.35, while 9% of triplets have an O-information score of below −0.5, and only 0.4% have a strongly synergistic score of below −0.7. Similar trends are seen in the YFS data, as shown in Figure 1b,d.

### 4.2. Predicting Synergy from Lower-Order Features

For predicting the PID-based synergy scores, the recursive feature selection process for the YFS dataset retained the following variables: Mutual Information Sum, Mutual Information Spread, Entropy Spread, Spearman Correlation, Kendall Correlation, Chi-Squared, Minimum Mutual Information, Maximum Mutual Information, Variation of Information, Normalized Variation of Information, Information Overlap, Normalized Information Overlap, Jensen-Shannon Divergence for three variables, and Co-Inertia. For the UKB dataset, the same variables were retained, with the addition of Average Entropy.

For the UKB dataset, the recursive feature selection process focusing on O-information retained the following variables: Mutual Information Sum, Mutual Information Spread, Minimum Mutual Information, Maximum Mutual Information, Custom Dual Entropy, Variation of Information, Normalized Variation of Information, and Information Overlap. Similarly, the YFS dataset retained these variables along with Kendall’s tau between x and z, Normalized Information Overlap, and Jensen–Shannon Divergence. The custom Dual Entropy was calculated as the average of the sum of the entropies of each variable minus the entropies of their pairwise combinations.

**Figure 2:**
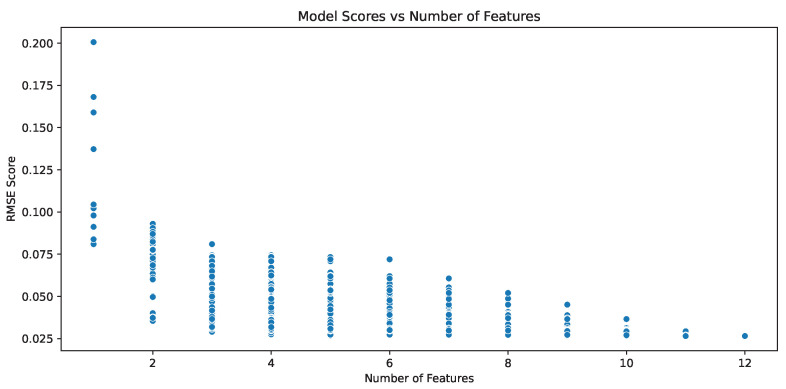
RMSE scores of the LightGBM model for predicting O-information scores based on different combinations of pairwise features. This plot illustrates the relationship between the number of features and the model’s prediction accuracy for O-information on the UKB NMR data. As expected, increasing the number of features generally leads to improved model accuracy, resulting in lower RMSE scores. A similar trend was found for both synergy metrics and both datasets.

Consequently, we used combinations of these variables in our regression analysis to identify the optimal pairwise feature combinations for our search algorithms. The analysis of the relationship between the number of features and the Root Mean Squared Error (RMSE) scores, as depicted in Figure 2, reveals a clear trend of decreasing RMSE with an increasing number of features. This indicates that models incorporating a greater number of pairwise features generally exhibit improved predictive accuracy. Notably, certain combinations significantly outperform others with a large variability in RMSE scores for each feature count. The models with the best predictive power are observed with the inclusion of approximately 9 to 12 features, where the RMSE scores reach their minimum values.

The best model achieved an RMSE score of 0.015 and 0.009 for predicting O-information and PID-based synergy scores in the UKB NMR data. Table A1, Table A2, Table A3 and Table A4 show the scores when predicting the PID-based synergy with combinations of six or fewer pairwise metrics as training features from our model. This shows that combinations of pairwise metrics can indicate the strength of synergy within a set of variables. However, computing a vast array of pairwise features to predict synergy values does not immediately benefit our goal of quickly identifying synergy in datasets, as gathering the training data also suffers from the combinatorial explosion facing synergy estimators. Therefore, our objective is to identify a robust set of features that can be utilized in search algorithms as proxies to guide our search efficiently.

Based on our analysis, if a regression model allowed, at most, six feature combinations, the optimal set included Mutual Information Sum, Mutual Information Spread, Minimum Mutual Information, Maximum Mutual Information, Variation of Information, and Normalized Variation of Information, achieving the lowest RMSE of 0.0169. If only two features were used, the best pair was Mutual Information Spread and Information Overlap, with an RMSE of 0.0524. Among individual variables, Mutual Information Sum was the most effective, with an RMSE of 0.0657. Additionally, the combination of Mutual Information Sum and Mutual Information Spread had an RMSE of 0.0556, while Minimum Mutual Information alone had an RMSE of 0.0674. As the top performing combinations rely on variants of mutual information and conditional entropy, these are the pairwise measures that are included in our subsequent search strategies.

### 4.3. Reducing the Number of Synergistic Sets to Analyse: Remove Triplets After Computing Synergy

**Figure 3:**
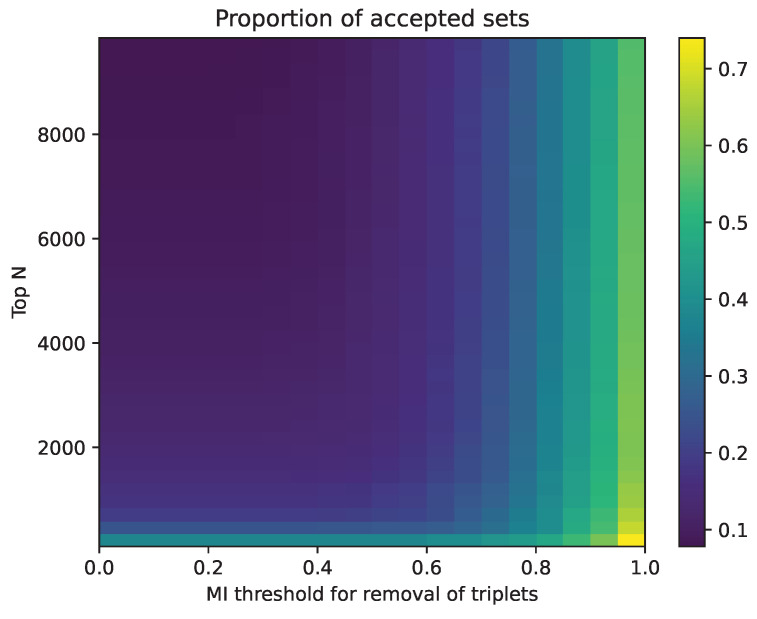
Prevalence of echoes in UKB NMR. If we select the top N synergistic sets in the data (*y*-axis) and then set an MI threshold for similarity (*x*-axis), the plot shows the proportion of synergistic sets that would remain after filtering. Most applications would lie in the bottom right of this figure.

In order to reduce the number of triplets to be analysed, we ran a naive heuristic on the n most synergistic triplets in the UKB NMR dataset, calculated using the O information, to identify and remove possible echo triplets. The filtering process involved iteratively selecting the most synergistic triplet and finding all other triplets with two common nodes. If the MI of the non-overlapping element was greater than p, then the less synergistic triplet was removed. This continued until all triplets had been checked. All accepted sets then have a high synergy and low mutual information between the triplets. Figure 3 shows a grid of different thresholds for the top number of triplets, ranging from 100 to 10,000 in increments of 250, and the mutual information thresholds varied from 0 to 1 in 20 steps. For each combination, the number of accepted and rejected sets was recorded, and matrices of the proportions of accepted and rejected sets were constructed. We can see that analysing the top 100 variables with a removal threshold of MI = 0.6 leads to the removal of 62% of the triplets, while analysing 1100 variables with the same threshold would remove 80% of the triplets. This leads to a massive reduction in the number of triplets that need to be analysed in hypernetworks, statistically tested, and interpreted by domain experts.

### 4.4. Reducing the Number of Synergistic Sets to Analyse: Remove Variables Before Computing Synergy

To reduce the computational burden of studying complex systems with a large number of variables, researchers often use feature selection methods to first reduce the number of variables to analyse. Methods such as Correlation-Based Feature Selection, Mutual Information-Based Feature Selection and Forward/Backward Selection often examine pairs of variables to determine which variables to remove before further analysis. However, we argue that it is crucial to account for all synergistic interactions without prematurely reducing the feature set. Performing the feature selection before computing the synergistic associations can overlook important synergies in the system.

**Figure 4:**
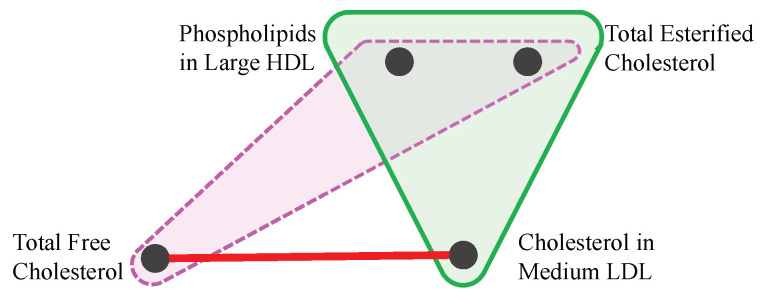
Example of a non-echo. *Total Free Cholesterol* and *Cholesterol in Medium LDL* have a strong MI score (red line). *Cholesterol in Medium LDL* forms a high synergy score with two other variables (green triangle). However, the triplet containing *Total Free Cholesterol* instead of *Cholesterol in Medium LDL* does not form a highly synergistic triplet (dashed purple triangle). This shows that if *Total Free Cholesterol* was used in the search for synergy, but *Cholesterol in Medium LDL* was not, then this synergistic association would be missed.

To illustrate this point, Figure 4 shows a set of four variables: *Cholesterol in Medium LDL*, *Total Free Cholesterol*, *Total Esterified Cholesterol*, and *Phospholipids in Large HDL*. Variables *Cholesterol in Medium LDL* and *Total Free Cholesterol* have a high mutual information score of 1.2, placing them in the 97.6th percentile of all MI scores. However, these variables exhibit different synergy values when combined with other variables: the triplet (*Cholesterol in Medium LDL, Total Esterified Cholesterol, Phospholipids in Large HDL*) is strongly synergistic in the data with a bias-corrected O-information score of −1.22, while the triplet (*Total Free Cholesterol, Total Esterified Cholesterol, Phospholipids in Large HDL*) has a very weak synergy score of −0.16.

**Figure 5:**
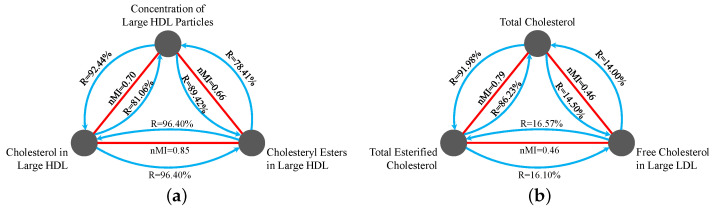
Examples of highly correlated sets of variables in the UKB NMR data. For each highly synergistic triplet (defined as having less than twice the mean O-information synergy score) containing each variable, we substitute an element from the triplet with a highly correlated variable from this set, and record the percentage of triplets that remain highly synergistic, as indicated by the percentages in the curved blue lines. The arrow on the curved blue lines indicates that the variable (one of the highly correlated variables) in the highly synergistic triplets is replaced with the other variable in the highly correlated pair. Normalized mutual information (nMI) scores are depicted by the red straight lines. (**a**) illustrates a case where three variables exhibit strong pairwise associations and share similar synergies, while (**b**) depicts variables with strong pairwise associations but very different synergies, as indicated by the low R scores. (**a**) Set of triplets with mostly the same synergies. (**b**) Set of triplets with differing synergies.

To further illustrate the danger of removing variables before assessing their synergies, we investigated the O-information synergies in the UKB NMR data to find highly correlated variables with different synergies. For a set of highly correlated variables, we assessed the robustness of the highly synergistic triplets by substituting one element from each synergistic triplet with a highly correlated variable. The results, shown by the percentages in the curved blue lines in Figure 5b, indicate how often the triplets remain highly synergistic after the substitution. Considering a highly correlated pair of variables, if the number of highly synergistic triplets that contain one of the variables in the pair is *N*, and after substitution, the number of triplets that remain highly synergistic is *k*, then the percentage is k/N∗100%.

Notably, Figure 5a depicts a scenario where three variables have strong pairwise associations and generally maintain their synergies. In contrast, Figure 5b demonstrates a case where the variables, despite having strong pairwise associations, exhibit very different synergies, as evidenced by their low R scores. This analysis underscores the importance of retaining all variables to capture the full spectrum of higher-order associations in the system, rather than using univariate methods to reduce the size of the system. By retaining all relevant variables until the synergistic interactions are fully explored, we can uncover extra synergies that would otherwise remain hidden. This approach ensures a more comprehensive and accurate understanding of the underlying complexities in the system.

### 4.5. Clique Identification in Pairwise Network

**Figure 6:**
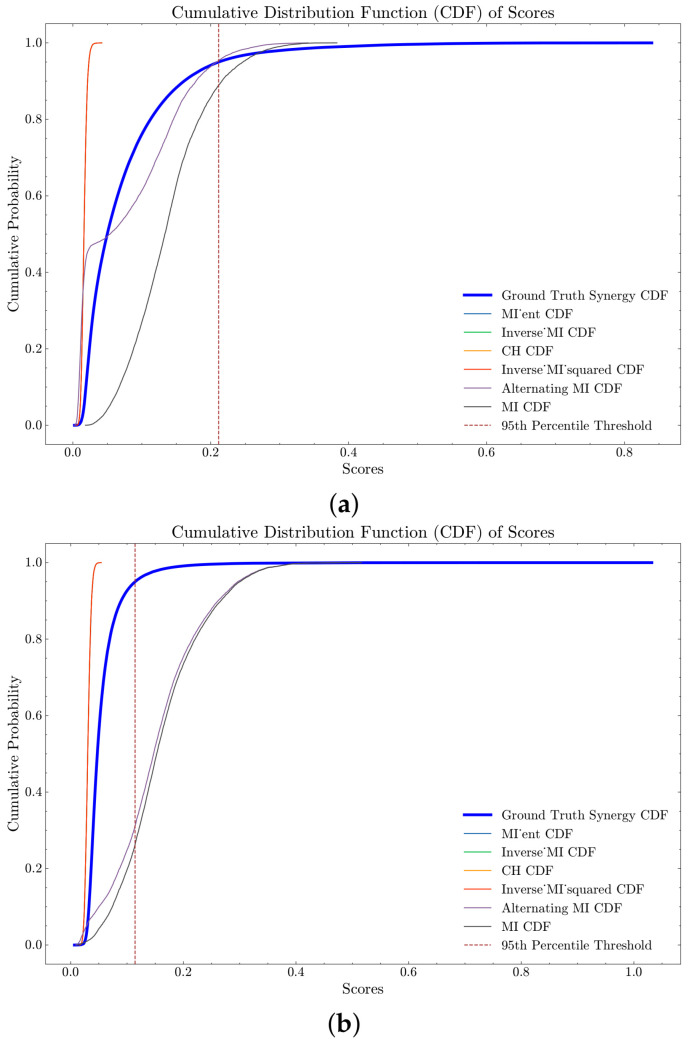
CDF of PID Scores for different pairwise metrics with the clique search strategy on UKB NMR and YFS datasets. (**a**) CDF of PID Scores for different pairwise metrics with the clique search strategy on UKB NMR. (**b**) CDF of PID Scores for different pairwise metrics with the clique search strategy on YFS.

**Figure 7:**
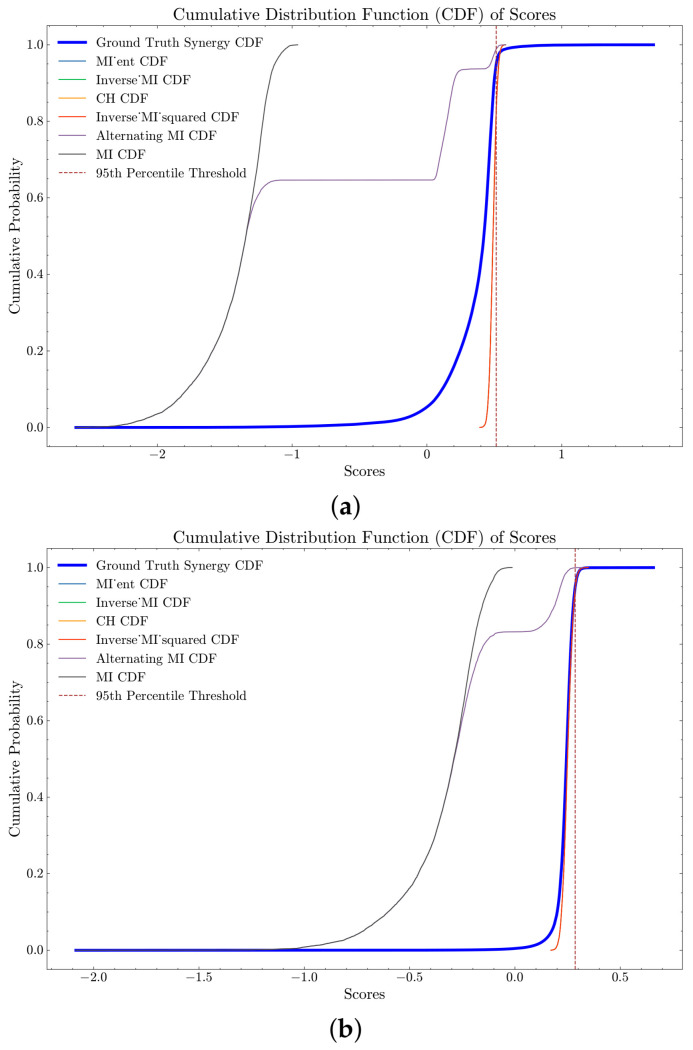
CDF of negated O-information Scores for different pairwise metrics with the clique search strategy on UKB NMR and YFS datasets. These plots compare the performance of each strategy against the ground truth synergy scores, with the ground truth CDF highlighted by a thicker blue line for distinction. Percentile thresholds are indicated by dashed lines. Better performance is represented by curves that are initially below and to the right of the solid blue line. (**a**) CDF of negated O-information scores for different pairwise metrics with the clique search strategy on UKB NMR. (**b**) CDF of negated O-information scores for different pairwise metrics with the clique search strategy on YFS.

In our analysis, we compared the performance of different pairwise metrics in the clique search strategy using the UKB NMR and YFS datasets. The results are presented in Figure 6 and Figure 7, which show the Cumulative Distribution Functions (CDFs) of PID and negated O-information synergy scores for each strategy. The ground truth synergy scores are highlighted with a thicker blue line, and percentile thresholds are marked with dashed lines. Curves that are initially below and to the right of the solid blue line indicate better performance, demonstrating the effectiveness of the respective strategies in identifying highly synergistic variable sets. We see that performance of different pairwise metrics vary depending on the synergy metric used: PID-based synergy metrics perform similar across datasets (Figure 6a,b) as do O-information based synergies (Figure 7a,b).

For the UKB NMR dataset PID synergy scores, the strategies *MI ent* (computed as $\left(H_{XYZ}⊙e^{I_{XYZ}^{2}}\right)$), *Inverse_MI*, *CH*, and *Inverse MI squared* displayed identical performance metrics with a mean score of approximately 0.0164, a median score of 0.0161, a standard deviation of approximately 0.0039, and an interquartile range (IQR) of around 0.0051. Notably, none of the scores from these strategies surpassed the 50th percentile of the ground truth scores, indicating limited effectiveness in identifying high-synergy sets. The Alternating MI strategy (motivated by the *MI spread* in the regression analysis) exhibited a higher mean score of approximately 0.0768 and a median score of 0.0541, with a higher standard deviation of 0.0723 and an IQR of approximately 0.1226. This strategy performed better, with 50.63% of its scores above the 50th percentile of the ground truth, though its effectiveness decreased at higher percentiles. The *MI* strategy outperformed all others with a mean score of approximately 0.1381, a median score of 0.1334, a standard deviation of 0.0577, and an IQR of 0.0738. Impressively, 95.86% of *MI* scores were above the 50th percentile, and 11.08% above the 95th percentile of the ground truth scores, highlighting its superior performance in identifying high-synergy sets.

Similarly, for the YFS dataset, *MI ent*, *Inverse MI*, *CH*, and *Inverse MI squared* had minimal scores exceeding the percentiles of the ground truth, indicating limited effectiveness. The Alternating MI strategy again demonstrated superior performance, with 90% of its scores above the 50th percentile of the ground truth and 68.86% above the 95th percentile. The *MI* strategy again showed superior performance with a mean score of 0.1622, a median score of 0.1526, a standard deviation of 0.0736, and an IQR of 0.0928. Notably, 96.40% of MI2 scores were above the 50th percentile, and 74% above the 95th percentile of the ground truth scores. The exact scores can be seen in Table A7, Table A8, Table A9 and Table A10.

The analysis using the O-information metric across both the UKB NMR and YFS datasets revealed significant performance differences among the various scoring strategies, as shown in Table A11, Table A12, Table A13 and Table A14. For the UKB NMR dataset, the strategies *MI ent*, *Inverse MI*, *CH*, and *Inverse MI* squared showed similar performance, with 99% of their scores exceeding the 50th percentile of the ground truth. However, their effectiveness diminished at higher percentiles, with only around 17.34% of their scores surpassing the 95th percentile. In the YFS data, these strategies had 59.43% of their scores above the 50th percentile, and about 5.52% above the 95th percentile, indicating a much more moderate effectiveness. The Alternating MI strategy had only 6.30% and 1.37% of its scores above the 50th percentile in the UKB NMR and YFS datasets, respectively, while none of the MI strategy scores exceeded the 50th percentile. The difference in performance is likely due to the YFS data having a differing number of states per variable, while the UKB NMR data has the same number of states across variables. After the pairwise metric is computed (brute-force) for the dataset, the algorithm has an average runtime of 240 s when searching for 10,000 triangles, using the same computer as in the PSO section.

### 4.6. Simulated Annealing for Synergistic Associations

Our approach expands upon the methodology presented in [22] by incorporating non-uniform probability distributions for selecting new node sets. Unlike the approach in [22], where replacement nodes are chosen randomly—a method we describe as ‘no weight’ and associate with the geometric cooling strategy—we introduce three alternative variants. These variants are designed based on the probability distribution of potential replacement nodes, defined as follows:

1. Using a probability distribution $1-\frac{MI}{\max(MI)}$.
2. Using mutual information directly.
3. Using $H_{XYZ}⊙e^{I_{XYZ}^{2}}$.

In the third variant, ⊙ signifies element-wise multiplication between matrices, $H_{XYZ}$ represents a vector containing the entropy of individual variables, and $e^{I_{XYZ}^{2}}$ signifies the exponentiated matrix of pairwise mutual information values. These features were derived using the results from our pairwise prediction models. This composite metric is constructed to synergize the mutual information and entropy, aiming to yield a high score when there are large entropy values and comparatively lower mutual information figures. This formulation ensures that neither entropy (*H*) nor mutual information (MI) disproportionately influences the overall score.

**Figure 8:**
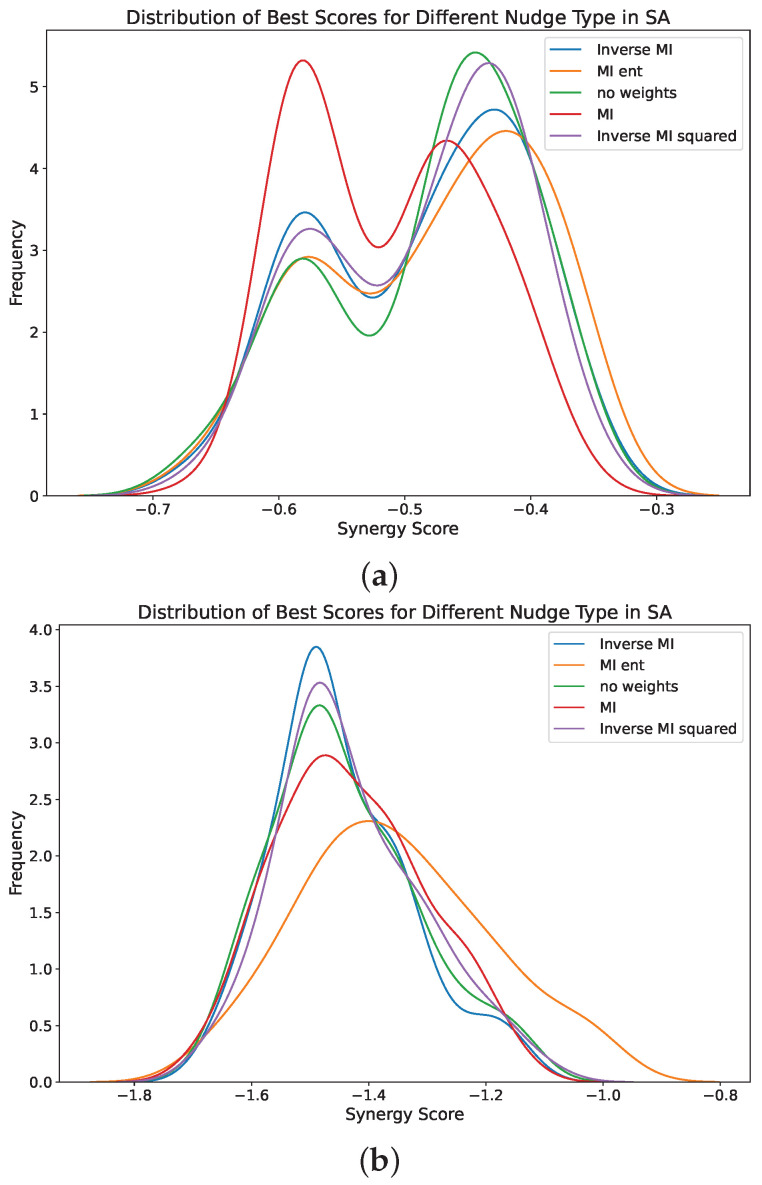
Distribution of best synergy scores found in the YFS and UKB NMR data for 100 runs of the SA algorithm using different strategies to guide particle movement. Synergy was computed via the O-information. The SA algorithm used a geometric cooling strategy with random initializations for each run. Lower values (to the left) indicate triplets with more synergistic information were found. Each particle movement strategy converged multiple times to triplets in the overall top 100 synergistic triplets in each dataset. (**a**) Distribution of best synergy scores found in the YFS data for 100 runs of the SA algorithm. (**b**) Distribution of best synergy scores found in the UKB NMR data for 100 runs of the SA algorithm.

The analysis of different SA configurations for the discretized NMR UKB dataset revealed significant performance differences among various scoring strategies, as shown in Table 1. The configurations Inverse MI geometric and no weights geometric demonstrated the most reliable performance, achieving the lowest mean and median scores with a high count of runs below the threshold. Specifically, Inverse MI geometric achieved a mean score of −1.4469, a median score of −1.4803, and had 68 runs below the threshold. Similarly, no weights geometric had a mean score of −1.4403, a median score of −1.4803, and 66 runs below the threshold. The MI ent geometric configuration showed significantly poorer performance, with a mean score of −1.3556, a median score of −1.3671, and only 42 runs below the threshold. Statistical tests further confirmed that MI ent geometric was significantly different from the other configurations, which performed similarly to each other, as seen in Figure 8.

The results from the SA algorithm using the Inverse MI nudge type and Boltzmann cooling strategy indicate a notable difficulty in consistently finding highly synergistic triplets. This challenge is evidenced by the frequent instances where the SA algorithm appears to become stuck in suboptimal solutions, as depicted in the first figure. However, when the SA algorithm does identify a highly synergistic triplet, it converges appropriately, as shown in subsequent figures. This behaviour suggests that while the SA algorithm can perform well under certain conditions, it often struggles with escaping local optima, and requires further refinement to enhance its reliability and consistency.

The performance of various SA configurations in identifying the most synergistic sets on the UKB NMR data using the O-information metric is summarized in Table 1. Similar results for the YFS data are shown in Table A15. All configurations exhibited similar mean and median best scores. Notably, the inverse MI and no weights configurations demonstrated narrower interquartile ranges (IQR), indicating more consistent results compared to other approaches. Additionally, a synergy threshold was set to evaluate how frequently each SA run identified a highly synergistic set among the 107 global best sets. While all configurations consistently reached this threshold, the MI ent strategy did so less frequently. These results suggest that the SA algorithms can effectively estimate the most synergistic triplets without exhaustive computation or extensive random sampling.

The training trajectories of the SA, as shown in Figure A4, often varied in their performance across different runs. The optimization landscape appears to be relatively flat, leading some runs to quickly find optimal solutions, while others become trapped in local minima. Consequently, running SA multiple times is necessary to ensure highly synergistic sets are found. As the particle moves in the SA search, it may sometimes accept a worse solution. However, returning to the better solution appears not always to be possible because of this difficult landscape. To address this challenge, employing a PSO algorithm could be advantageous, as it allows for the swarm of particles to collaboratively explore the search space, potentially enabling quicker escapes from flat regions and always nudging particles back toward their local optima, further improving the overall search efficiency.

The SA algorithm was executed 100 times for each parameter setting, with each run comprising 1000 samples. The running time for completing these runs varied. The running times ranged from a minimum of approximately 30 min to a maximum of approximately 57 min per complete set of runs for a given parameter setting. The simulations were run on a laptop with an Intel64 Family 6 Model 154 Stepping 4, GenuineIntel processor, 10 physical cores, max Frequency of 1700.00Mhz and RAM of 31.64 GB. Multiprocessing will further enhance running times when deploying this algorithm on larger datasets.

### 4.7. Particle Swarm Optimization

The performance of the PSO algorithm was evaluated using different parameter settings for nudge and weight types. Figure A5 and Figure A6 show the convergence rates of the PSO algorithm for various configurations. The plots demonstrate how the global best score changes over time, highlighting the effectiveness of different nudge strategies and weight types, where the same weight types were used as in the SA algorithm. It is evident that certain configurations lead to faster and more consistent convergence compared to using no nudges. However, there does not appear to be a significant difference in performance between nudge types.

Additionally, Figure A3 presents the initial and final scores for each particle in the PSO algorithm for a specific parameter setting. This figure illustrates that the PSO algorithm successfully guides particles towards better solutions, as evidenced by the significant improvement in scores from start to end. Unlike the SA approach, where particles often become stuck in local minima, the PSO algorithm effectively explores the search space, ensuring more particles reach optimal or near-optimal solutions.

### 4.8. Synergistic Information Patterns in Lipid Metabolism

**Figure 9:**
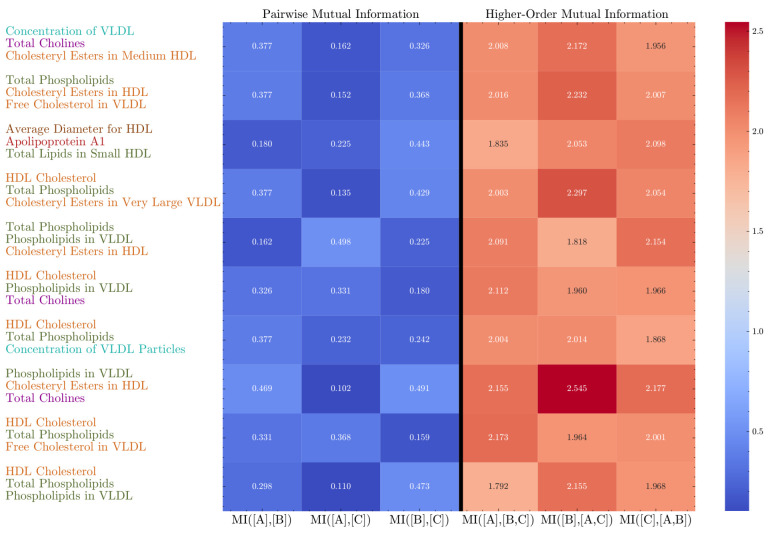
Heatmap of pairwise and higher-order mutual information (MI) for lipid metabolism triplets, with biological groupings. The heatmap displays the pairwise MI (columns 1–3) and the higher-order MI (columns 4–6) for each triplet, where variables are coloured based on their biological cluster. The strong synergies (higher-order MI) between variables from different clusters, such as HDL Cholesterol and Phospholipids in VLDL, highlight cross-functional interactions in lipid metabolism.

To further investigate the identified complex interactions among NMR metabolic metabolism biomarkers, we performed univariate and bivariate mutual information analyses on the top ten biomarker triplets with the lowest bias-corrected O-information (please see Appendix Table A5 and Figure A2 for the top ten triplets in terms of the largest improvement factors in the UK Biobank NMR dataset). These triplets spanned 13 unique biomarkers, as described in Table A6 and Figure 9, which were subsequently grouped (and coloured) based on their roles in cholesterol transport, lipoprotein particle concentration, phospholipids, and apolipoproteins. Each triplet combined biomarkers from one or more of these functional groups, providing a structured basis for interpreting biologically meaningful synergies.

**Figure A1:**
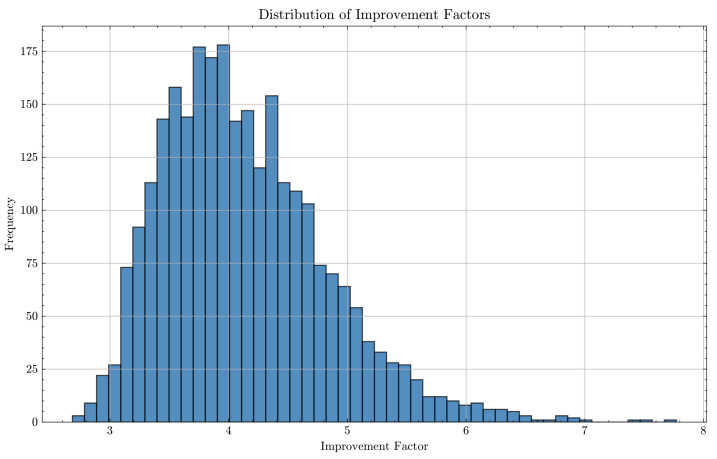
Distribution of improvement factors for triplet interactions. The improvement factor quantifies the ratio between the joint mutual information of two variables about the third and the sum of their pairwise mutual information. The histogram reveals that most triplets exhibit improvement factors between 3 and 5, with a peak around 4, indicating that synergistic interactions are common. The presence of a long tail with higher values suggests that some triplets exhibit particularly strong synergies, highlighting the importance of higher-order interactions in lipid metabolism.

**Figure A2:**
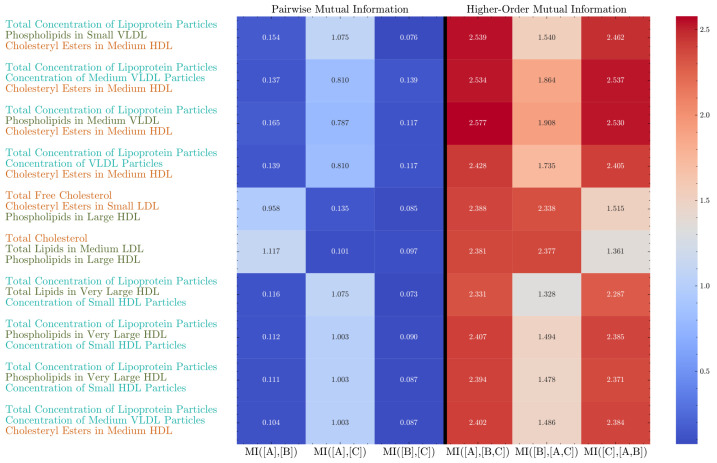
Heatmap of pairwise and higher-order mutual information (MI) for lipid metabolism triplets with significant synergy and large improvement factors, categorized by biological function. The heatmap presents both the pairwise MI (columns 1–3) and the higher-order MI (columns 4–6) for each triplet. Variables are colour-coded according to their biological cluster, showing that these triplets exhibit strong synergistic interactions and large improvement factors. The analysis reveals that interactions between variables from different clusters, such as lipoprotein particle concentration and cholesterol transport, exhibit stronger synergies, suggesting coordinated regulation of lipid transport and metabolism.

**Figure A3:**
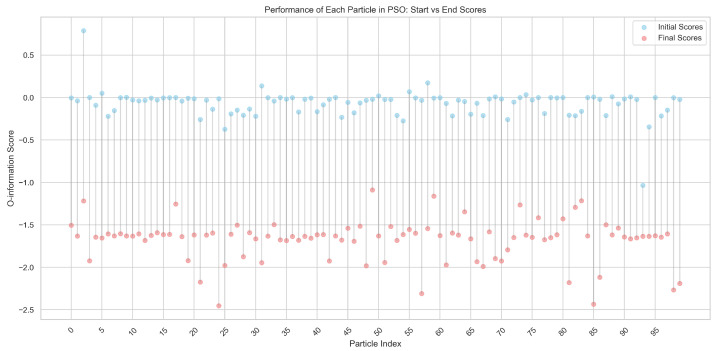
Initial and final scores for each particle in the PSO algorithm for one parameter setting. The plot shows that the particles generally improve their positions, moving towards lower O-information scores, indicating that the PSO algorithm effectively prevents particles from getting stuck in local minima compared to the SA approach.

**Figure A4:**
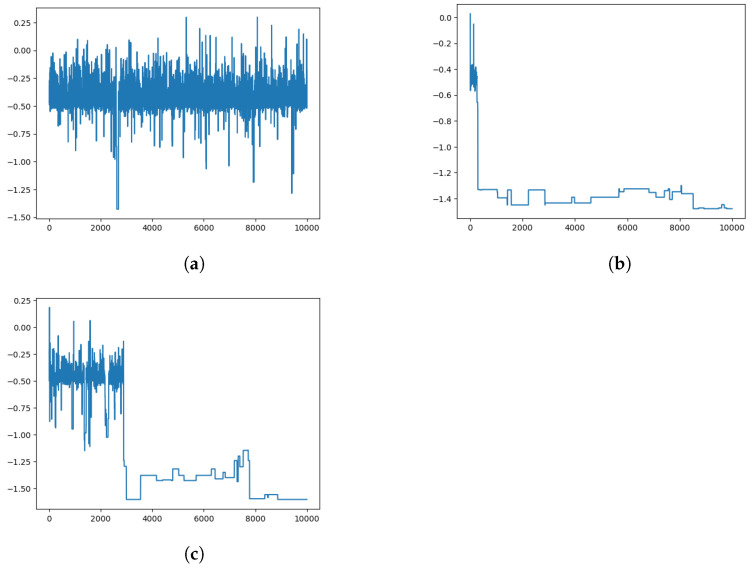
Training trajectories of three different runs of the SA algorithm. These plots illustrate the performance and challenges faced by the SA in navigating the optimization landscape. The variability in the trajectories indicates the algorithm’s sensitivity to initial conditions and potential difficulties in escaping local minima. (**a**) No significant improvement: In this plot, the SA algorithm remains trapped in a region of suboptimal solutions throughout the run, failing to find any highly synergistic triplets. The scores fluctuate around the same range, indicating that the algorithm is unable to escape local minima in this particular run. (**b**) Slow initial improvement: This plot demonstrates a scenario where the SA algorithm initially struggles to find a good solution, resulting in high variability in scores. However, after approximately 4000 steps, the algorithm starts to improve steadily, eventually finding a highly synergistic triplet. (**c**) Quick improvement: this plot shows a trajectory where the SA algorithm rapidly finds a highly synergistic triplet within the first few thousand steps and maintains the low score with minor fluctuations.

**Figure A5:**
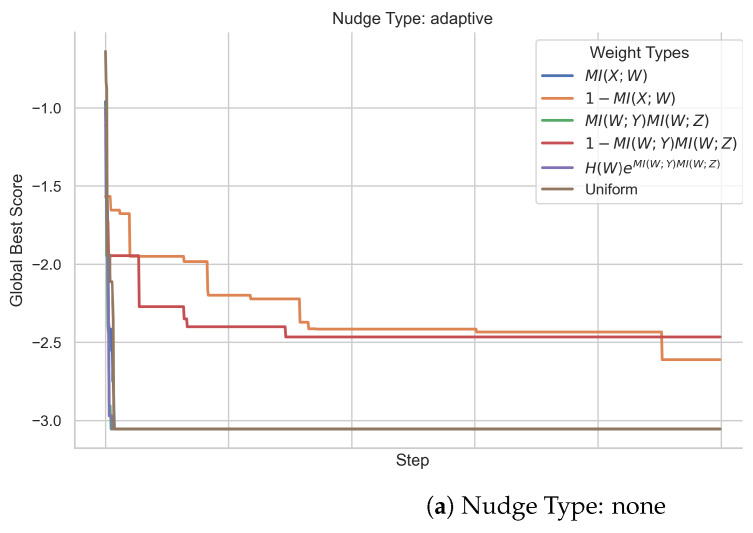

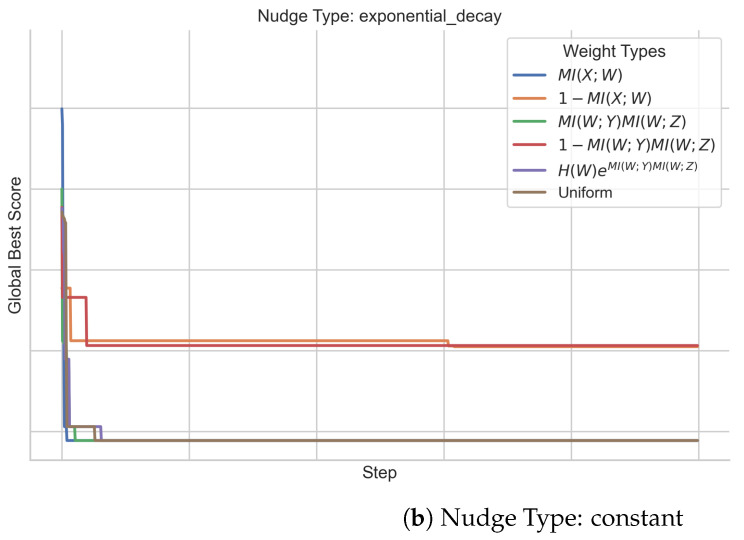
Convergence rates of the PSO algorithm with nudge types none and constant. The plots illustrate how the global best score evolves over time for various weight types used in the SA algorithm.

**Figure A6:**
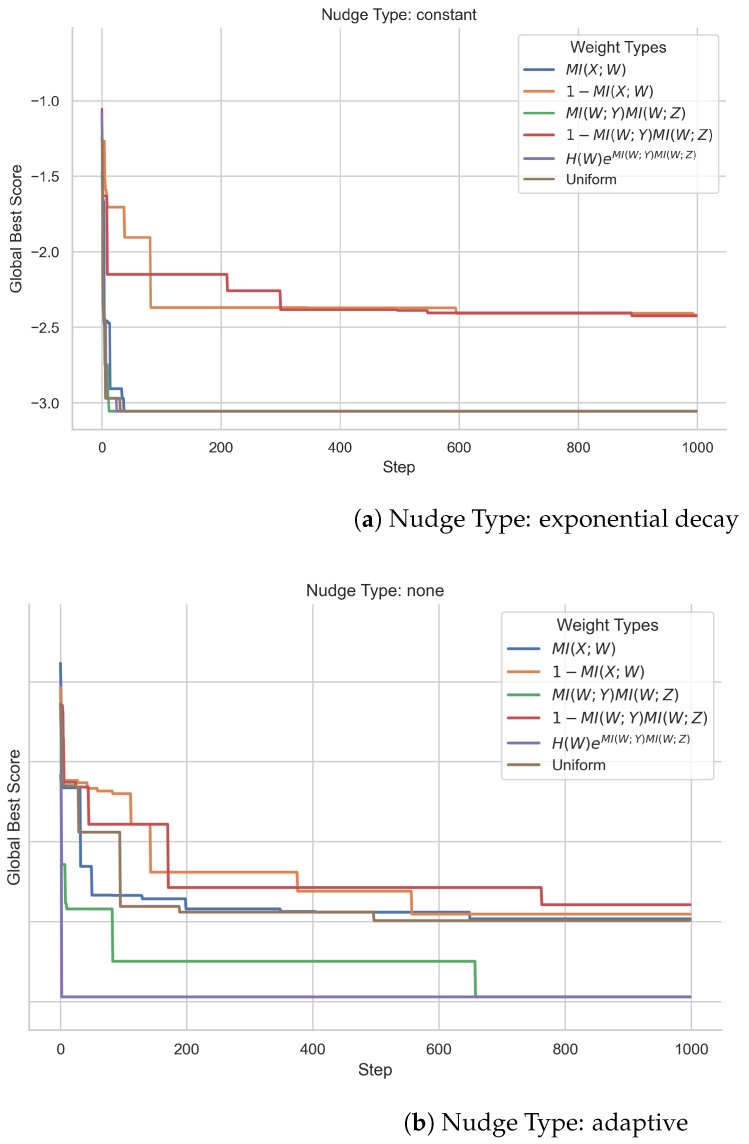
Convergence rates of the PSO algorithm with nudge types exponential decay and adaptive. The plots illustrate how the global best score evolves over time for various weight types used in the SA algorithm.

We recall that the improvement factor quantifies the extent to which the joint MI of two variables about the third exceeds the sum of their individual pairwise MI values. The distribution of improvement factors across strongly synergistic triplets (taken to be O-information scores below −0.4) is shown in Figure A1. We observe that most factors are between 3 and 5, with a peak around 4 (mean 4.13, std 0.69). This indicates that, on average, the joint interaction between two variables provides roughly four times more information about the third variable compared to pairwise associations. In other words, if one would focus on only pairwise associations, then in these triplets we would miss about 75% of the explanation (reduced conditional entropy) of each variable. The presence of a long tail extending toward values up to 8 highlights the existence of certain triplets with exceptionally strong synergies, suggesting complex, biological interactions that are not captured at all by pairwise MI. This highlights the added value of our analysis.

**Figure 10:**
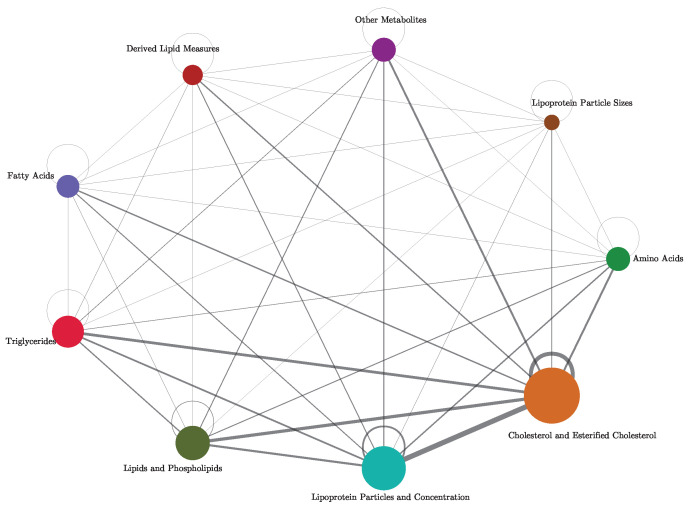
Network visualization of synergistic interactions between metabolite categories. Each node represents a category of metabolites, and the size of each node reflects the number of variables in the UKB NMR data assigned to that category. Edge thickness represents the number of synergistic triplets between pairs of categories, with thicker edges indicating more synergistic associations between those variable categories. Node colours correspond to distinct categories: Amino Acids, Lipoprotein Particle Sizes, Other Metabolites, Derived Lipid Measures, Fatty Acids, Triglycerides, Lipids and Phospholipids, Lipoprotein Particles and Concentration, and Cholesterol and Esterified Cholesterol. Self-loops, indicating within-category synergy, are positioned outside the nodes to enhance clarity.

Cross-referencing these results with the variable groups, we find that triplets involving biomarkers from different functional groups tend to exhibit stronger synergies than those from the same group, as shown in the network of Figure 10. An edge is drawn in the network when a synergistic triplet contains variables from both categories, where the edge weight shows the number of times this occurs. All edges are present in the network, indicating that synergy is present between all variable categories. This indicates that triplets comprising biomarkers from distinct functional groups exhibit strong synergies between groups.

However, it is interesting to note that the most synergistic variables, as shown in Figure 9, only have variables from six of nine categories. For example, as shown in row 10 of Figure 9, the triplet of HDL Cholesterol, Total Phospholipids, and Phospholipids in VLDL demonstrates moderate pairwise MI values, yet a substantial higher-order MI, highlighting a significant synergy between these variables. The high synergy here may reflect a coordinated regulation between HDL and VLDL particles, potentially impacting lipid transport and cholesterol homeostasis. Notably, the combined information of HDL Cholesterol and Total Phospholipids about Phospholipids in VLDL far exceeds the sum of their pairwise MI values, suggesting that these interactions might involve mechanisms such as phospholipid exchange between HDL and VLDL, which helps to maintain lipid balance. Similarly, the triplet including the Average Diameter of HDL, Apolipoprotein A1, and Total Lipids in Small HDL exhibited low pairwise MI values but revealed a high improvement factor when including Average Diameter, indicating interactions potentially related to HDL size, cholesterol efflux capacity, and functionality.

### 4.9. Plausible Mechanistic Interpretations

To further our qualitative analysis, we propose speculative interpretations of selected synergistic triplets based on current knowledge of lipid metabolism. These interpretations are intended to assess the biological plausibility of our findings, providing insights into potential functional relationships, although they do not serve as definitive mechanistic conclusions.

Another strong synergy is observed in the triplet containing the Average Diameter of HDL, Apolipoprotein A1, and Total Lipids in Small HDL. In this triplet, the pairwise MI between Apolipoprotein A1 and Total Lipids in Small HDL is very low (MI = 0.102). However, when the Average Diameter is introduced as a third variable, the higher-order MI increases substantially (MI = 2.545), indicating a strong synergistic interaction. This hints at a complex mechanism by which two of the variables modulate the third. In this case, HDL particles are heterogeneous in size and composition, with ApoA1 serving as the primary structural protein [28]. More specifically, ApoA1 forms a scaffold-like structure that encapsulates lipids in HDL particles. Varying the number of ApoA1 molecules per particle would affect both particle size and lipid capacity. Thus, the relationship can be explained by ApoA1 acting as a modulator: the amount of ApoA1 available can modulate the relationship between HDL size and lipid content. In other words, more ApoA1 allows for larger particles that can accommodate more lipids. Another process acting in parallel in this example is that the lipid content influences the size of the HDL particles as well. That is, even if there is ample ApoA1 available in the environment, there is another process that can explain the synergy. Namely, ApoA1 has two conformational states (large and small), and the amount and types of lipids associated with ApoA1 can cause conformational changes as the protein adapts to encapsulate the different lipid quantities. Although, of course, many more biological processes happen in parallel that may affect the information synergy that we eventually measure, these insights give us confidence that what we are measuring is indeed meaningful and insightful. In cases where there is not yet (or not yet sufficient) biological mechanistic explanations available, our results may even give direction to further investigation into such higher-order effects. As an aside, this example also shows how three different functional groups can act together to create complex, non-linear mechanisms.

One notable triplet—Total Lipoprotein Particle Concentration, Medium VLDL Particle Concentration, and Cholesteryl Esters in Medium HDL—demonstrated moderate pairwise MI values, but an improvement factor of 6.2. One plausible explanation is that an increase in total lipoprotein particles could indicate elevated lipid flux, potentially triggering increased VLDL production by the liver. This rise in VLDL particles would provide more acceptors for cholesteryl esters transferred from HDL via Cholesteryl Ester Transfer Protein (CETP) activity. So far, the process is linear. However, simultaneously, the heightened lipid flux might also stimulate reverse cholesterol transport, which increases cholesteryl ester content in medium HDL. The transfer of these esters from HDL to VLDL would tend to decrease HDL cholesteryl ester content. This happens during a process of lipolysis (making lipids smaller): as VLDL particles undergo lipolysis, they interact with HDL particles, exchanging lipids and apolipoproteins [29]. Additionally, this has a ‘non-linear interaction’ with the way the data are organized, as the ‘Medium VLDL’ variable would first increase, but then decrease again during this process, while the decreased state is actually ambiguous. That is, if ‘Medium VLDL’ is low, then the VLDL could be predominantly large or small, which are different biological states that are measured by different variables in the data set, which were not part of this triplet (such interactions may in fact become clear when analysing quadruplets, quintuplets, etc.). This complex, non-linear interaction between lipoprotein production, CETP-mediated lipid transfer, and reverse cholesterol transport could explain the high multivariate correlation, despite low pairwise correlations.

## 5. Discussion

The study of higher-order dependencies in multivariate data has seen significant growth, emphasizing the need to identify synergistic sets. Traditional heuristics for computing synergy often face scalability issues, necessitating the development of innovative approaches for efficient search space navigation.

Our novel methodology employs stochastic strategies SA and PSO, alongside a direct network-based clique algorithm, to identify synergistic triplets. These methods alleviate the computational burden inherent to exhaustive searches, proving highly effective when applied to datasets with a vast number of variables. The integration of lower-order statistical features into the search algorithms enhances their ability to predict top synergistic associations with high accuracy.

Our results demonstrate that, while SA can identify highly synergistic triplets, it often becomes trapped in local minima, requiring multiple runs to ensure comprehensive exploration. Conversely, the PSO algorithm, through its collaborative nature, prevents particles from getting stuck in local minima, thus achieving more robust results. The clique-based heuristic algorithm, though faster, serves as a preliminary assessment tool rather than a definitive method for synergy identification.

Additionally, our study addresses the “echo problem”, which arises when strong pairwise associations are connected to synergistic triplets, leading to an overestimation of synergistic sets. Specifically, if a strong pairwise association exists between two variables, it is likely that they can be interchanged in different synergistic triplets. This can lead to an explosion of identified triplets to analysis, many of which convey the same information. The echo problem significantly impacts the global network structure, altering node centrality, path length distributions, and clustering, which can mislead downstream analysis. We propose a naive regularization step incorporated into the global optimization process to filter out these spurious associations. By ensuring that only truly synergistic sets are retained, we improve the reliability and interpretability of the results.

Furthermore, we caution against removing variables before computing the synergy, as this could result in missing important information. Highly correlated variables often exhibit different synergies with other variables, and their removal could lead to the omission of critical synergistic interactions. We present examples demonstrating that retaining all variables until the synergistic interactions are fully explored allows for the discovery of additional synergies that would otherwise remain hidden. This approach ensures a more comprehensive and accurate understanding of the underlying complexities in the system.

Our analysis underscores the complex regulatory network within lipid metabolism, with significant synergies observed across different lipoprotein classes. While the precise mechanisms remain speculative, our findings suggest that higher-order interactions among lipid-related biomarkers could be integral to lipid transport and homeostasis. These interpretations set a foundation for future empirical studies to explore the observed synergies and validate their roles in lipid metabolism.

Future research should extend this approach to larger sets, explore additional synergy measures, and compute synergy at different orders beyond triplets. Further exploration of heuristic seeding for search algorithms and network community detection could enhance the efficiency and robustness of these methods.

## 6. Conclusions

From our study, we conclude that identifying significant and highly ranking synergistic triplets in large datasets is feasible using stochastic search algorithms. While a brute-force approach on our datasets requires about one week of continuous computation (single core), the SA and PSO algorithms take approximately two to three hours. The PSO algorithm outperforms SA in terms of robustness, and while both strategies are effective in finding rare, highly synergistic triplets, the heuristic algorithm is even faster, providing an initial assessment of synergies, but a search algorithm follow-up is necessary for trustworthy results.

In future work, we plan to explore whether seeding search algorithms with triplets identified by the heuristic algorithm can speed up the search without leading to local optima or excessive particle collisions. Thus, we expect that for such a heuristic seeding some additional procedure is required to prevent clustering of particles. One option could be to first perform a network community detection, and then execute the heuristic algorithm per community, but there are many details to be explored such as preventing communities from becoming too small, but also the question of how many (and which) synergistic triplets would no longer be found due to splitting the network.

The echo problem, where strong pairwise associations lead to the identification of redundant synergistic triplets, underscores the importance of a careful regularization process. By addressing this issue, we can ensure that the identified synergies are both meaningful and non-redundant, enhancing the reliability of the results.

In conclusion, our stochastic search strategy represents an advancement in the analysis of higher-order dependencies in multivariate data. It offers a scalable, efficient tool for uncovering complex interactions within large datasets. The inclusion of all relevant variables until the synergistic interactions are fully explored ensures a comprehensive understanding of the underlying complexities in the system. This approach mitigates the risk of missing out on critical synergies that could be overlooked by premature variable elimination, thus providing a more accurate and holistic view of the data.

## Figures and Tables

**Table 1:** Summary statistics for the best synergistic sets found by different SA configurations on the UKB NMR data using O-information. All approaches have similar mean and median best scores. The interquartile range (IQR) for the inverse MI, and the no weights setups are narrower than other approaches, indicating more consistent results. We also set a synergy threshold to determine how many times each SA run found a synergistic set in the 107 global best. All approaches consistently reached this threshold, although the MI ent strategy did so less frequently than the others. Reaching the threshold indicates that the SA algorithms can accurately estimate the most synergistic triplet in the data without needing to brute-force calculate or randomly sample a large number of triplets.

Configuration	Mean	Median	Std Dev	IQR	Below Threshold
Inverse MI	−1.4469	−1.4803	0.1149	0.1398	68
MI ent	−1.3556	−1.3671	0.1612	0.2304	42
no weights	−1.4403	−1.4803	0.1238	0.1391	66
MI	−1.4315	−1.4554	0.1243	0.1569	62
Inverse MI squared	−1.4304	−1.4584	0.1221	0.1504	65

## Data Availability

The data utilized in this study were obtained from the UK Biobank and the Young Finns Study. Researchers wishing to use the UK Biobank data must apply directly to the UK Biobank and comply with their data access policies (https://www.ukbiobank.ac.uk/ accessed on 4 Novermer 2024). Similarly, data from the Young Finns Study can be accessed by contacting the study’s data management team and complying with their access policies (http://youngfinnsstudy.utu.fi/ accessed on 4 Novermer 2024).

## Appendix A

**Table A1:** Feature combinations and respective RMSE scores (part 1) of the lightgbm model for predicting PID-based synergy scores in the UKB Biobank data.

Features	RMSE
Mutual Information Sum, Mutual Information Spread, Minimum Mutual Information, Maximum Mutual Information, Variation of Information, Normalized Variation of Information	0.016931
Mutual Information Spread, Minimum Mutual Information, Maximum Mutual Information, Variation of Information, Normalized Variation of Information, Information Overlap	0.016965
Mutual Information Spread, Minimum Mutual Information, Maximum Mutual Information, Variation of Information, Information Overlap, Normalized Information Overlap	0.016986
Mutual Information Sum, Mutual Information Spread, Maximum Mutual Information, Variation of Information, Normalized Variation of Information, Information Overlap	0.016988
Mutual Information Sum, Mutual Information Spread, Minimum Mutual Information, Maximum Mutual Information, Variation of Information, Information Overlap	0.017115
Mutual Information Sum, Mutual Information Spread, Minimum Mutual Information, Maximum Mutual Information, Normalized Variation of Information, Information Overlap	0.017143
Mutual Information Spread, Minimum Mutual Information, Maximum Mutual Information, Normalized Variation of Information, Information Overlap, Normalized Information Overlap	0.017433
Mutual Information Sum, Mutual Information Spread, Minimum Mutual Information, Maximum Mutual Information, Variation of Information, Information Overlap, Normalized Information Overlap	0.017512
Mutual Information Spread, Minimum Mutual Information, Maximum Mutual Information, Variation of Information, Information Overlap, Normalized Information Overlap	0.017532
Mutual Information Sum, Mutual Information Spread, Maximum Mutual Information, Variation of Information, Information Overlap, Normalized Information Overlap	0.017534

**Table A2:** Feature combinations and respective RMSE scores (part 2) of the lightgbm model for predicting PID-based synergy scores in the UKB Biobank data.

Features	RMSE
Mutual Information Sum, Minimum Mutual Information, Maximum Mutual Information, Variation of Information, Normalized Variation of Information, Information Overlap	0.017590
Mutual Information Sum, Minimum Mutual Information, Maximum Mutual Information, Variation of Information, Information Overlap, Normalized Information Overlap	0.017590
Mutual Information Sum, Mutual Information Spread, Minimum Mutual Information, Maximum Mutual Information, Variation of Information, Normalized Variation of Information, Information Overlap	0.017601
Mutual Information Sum, Mutual Information Spread, Minimum Mutual Information, Maximum Mutual Information, Variation of Information, Normalized Variation of Information, Information Overlap	0.017616
Minimum Mutual Information, Variation of Information, Normalized Variation of Information, Information Overlap, Normalized Information Overlap	0.017633
Mutual Information Sum, Minimum Mutual Information, Maximum Mutual Information, Normalized Variation of Information, Information Overlap, Normalized Information Overlap	0.017676
Minimum Mutual Information, Maximum Mutual Information, Normalized Variation of Information, Information Overlap, Normalized Information Overlap	0.017677
Mutual Information Sum, Minimum Mutual Information, Maximum Mutual Information, Variation of Information, Information Overlap, Normalized Information Overlap	0.017678
Mutual Information Spread, Minimum Mutual Information, Maximum Mutual Information, Variation of Information, Normalized Variation of Information, Information Overlap	0.017925
Mutual Information Sum, Mutual Information Spread, Minimum Mutual Information, Maximum Mutual Information, Variation of Information, Information Overlap, Normalized Information Overlap	0.017952

**Table A3:** Feature combinations and respective RMSE Scores (part 3) of the lightgbm model for predicting PID-based synergy scores in the UKB Biobank data.

Features	RMSE
Mutual Information Sum, Mutual Information Spread, Minimum Mutual Information, Maximum Mutual Information, Variation of Information, Normalized Variation of Information, Information Overlap	0.017992
Mutual Information Spread, Minimum Mutual Information, Maximum Mutual Information, Variation of Information, Information Overlap, Normalized Information Overlap	0.018010
Mutual Information Sum, Minimum Mutual Information, Maximum Mutual Information, Normalized Variation of Information, Information Overlap	0.053983
Mutual Information Spread, Information Overlap	0.054077
Mutual Information Sum, Mutual Information Spread	0.055605
Mutual Information Sum, Mutual Information Spread	0.055606
Mutual Information Spread, Variation of Information, Information Overlap	0.055612
Mutual Information Sum, Variation of Information, Information Overlap	0.059347
Mutual Information Sum, Avg_Corr	0.059360
Variation of Information, Avg_Corr	0.059373

**Table A4:** Feature combinations and respective RMSE Scores (part 4) of the lightgbm model for predicting PID-based synergy scores in the UKB Biobank data.

Features	RMSE
Maximum Mutual Information, Avg_Corr	0.059897
Mutual Information Spread, Avg_Corr	0.060684
Normalized Information Overlap, Avg_Corr	0.060902
Information Overlap, Avg_Corr	0.061232
Minimum Mutual Information, Normalized Information Overlap	0.061695
Minimum Mutual Information, Variation of Information, Normalized Information Overlap	0.062058
Mutual Information Sum, Minimum Mutual Information, Normalized Information Overlap	0.062059
Mutual Information Sum, Minimum Mutual Information, Normalized Information Overlap	0.062059
Minimum Mutual Information, Information Overlap	0.062973
Mutual Information Spread, Minimum Mutual Information	0.063003
Minimum Mutual Information, Maximum Mutual Information	0.063024
Mutual Information Spread, Minimum Mutual Information	0.063055
Mutual Information Spread, Maximum Mutual Information	0.063528
Mutual Information Sum	0.065725
Variation of Information	0.065725
Mutual Information Sum, Variation of Information	0.065725
Minimum Mutual Information	0.067377
Avg_Corr	0.067407
Maximum Mutual Information	0.067513
Mutual Information Spread	0.067699
Normalized Information Overlap	0.067820
Information Overlap	0.070113

### Appendix A.1. Biological Clustering and Synergistic Interactions in Lipid Metabolism

The following tables provide additional details on the biological clustering of lipid metabolism variables and the synergistic interactions among selected triplets with significant improvement factors. Each variable is categorized based on its biological function to give a high-level insight into the functional roles of each variable and support the interpretation of complex interactions within lipid transport.

Table A5 and Table A6 complement the main analysis by detailing how specific combinations of variables contribute to a more comprehensive understanding of lipid metabolism. The high improvement factors observed highlight instances where synergistic interactions provide more information than pairwise associations alone, emphasizing the importance of examining higher-order interactions to understand lipid homeostasis and transport mechanisms.

**Table A5:** Biological clustering of lipid metabolism variables in the selected triplets with significant synergy and large improvement factors. Each variable is categorized based on its biological function into one of three clusters: cholesterol transport and esterification, lipoprotein particle concentration and size, and phospholipids and lipid composition. These triplets were selected based on their high synergistic interactions and large improvement factors, which highlight how certain combinations of variables provide more information about lipid metabolism than individual pairwise associations. The grouping provides insight into the roles of the variables and helps interpret their complex interactions within lipid transport.

Variable Name	Description	Cluster/Grouping
Total Concentration of Lipoprotein Particles	The total number of lipoprotein particles across all lipoprotein classes.	Lipoprotein Particle Concentration and Size
Concentration of Medium VLDL Particles	The concentration of medium-sized VLDL particles, responsible for triglyceride transport.	Lipoprotein Particle Concentration and Size
Cholesteryl Esters in Medium HDL	Esterified cholesterol stored in medium-sized HDL particles.	Cholesterol Transport and Esterification
Phospholipids in Very Large HDL	Phospholipids found in very large HDL particles, important for structural integrity.	Phospholipids and Lipid Composition
Concentration of Small HDL Particles	The concentration of small HDL particles, important in cholesterol efflux.	Lipoprotein Particle Concentration and Size
Total Lipids in Very Large HDL	Total lipid content in very large HDL particles, contributing to lipid transport.	Phospholipids and Lipid Composition
Total Cholesterol	The total amount of cholesterol in all lipoprotein particles.	Cholesterol Transport and Esterification
Total Lipids in Medium LDL	Total lipid content in medium-sized LDL particles, involved in cholesterol transport.	Phospholipids and Lipid Composition
Phospholipids in Large HDL	Phospholipids found in large HDL particles, involved in cholesterol transport.	Phospholipids and Lipid Composition
Total Free Cholesterol	Unesterified cholesterol circulating in lipoproteins, available for uptake by tissues.	Cholesterol Transport and Esterification
Cholesteryl Esters in Small LDL	Esterified cholesterol stored in small LDL particles.	Cholesterol Transport and Esterification
Concentration of VLDL Particles	The concentration of VLDL particles, responsible for triglyceride transport.	Lipoprotein Particle Concentration and Size
Phospholipids in Medium VLDL	Phospholipids found in medium-sized VLDL particles, involved in lipid transport.	Phospholipids and Lipid Composition
Phospholipids in Small VLDL	Phospholipids found in small VLDL particles, important for triglyceride transport.	Phospholipids and Lipid Composition

**Table A6:** Biological clustering of lipid metabolism variables in the ten most synergistic triplets as per the bias-corrected O-information metric. Each variable is categorized based on its biological function into one of five clusters: cholesterol transport and esterification, lipoprotein particle concentration and size, phospholipids and lipid composition, apolipoproteins and small HDL function, or other metabolites. These groupings provide insight into the functional roles of the variables and help interpret their interactions in lipid metabolism.

Variable Name	Description	Cluster/Grouping
HDL Cholesterol	Cholesterol transported by HDL, involved in reverse cholesterol transport.	Cholesterol Transport and Esterification
Cholesteryl Esters in HDL	Esterified cholesterol in HDL, stored for transport to the liver.	Cholesterol Transport and Esterification
Concentration of VLDL Particles	Number of VLDL particles transporting triglycerides and cholesterol.	Lipoprotein Particle Concentration and Size
Total Phospholipids	Total amount of phospholipids in all lipoprotein particles.	Phospholipids and Lipid Composition
Cholesteryl Esters in Very Large VLDL	Esterified cholesterol in large VLDL particles, linked to lipid transport.	Cholesterol Transport and Esterification
Total Lipids in Small HDL	Total lipid content in small HDL particles, involved in cholesterol efflux.	Apolipoproteins and Small HDL Function
Apolipoprotein A1	Main protein component of HDL, critical for HDL formation and function.	Apolipoproteins and Small HDL Function
Concentration of VLDL	Concentration of VLDL particles, related to lipid transport.	Lipoprotein Particle Concentration and Size
Free Cholesterol in VLDL	Free, unesterified cholesterol in VLDL particles.	Other Metabolites
Total Cholines	Choline-containing compounds, precursors for phospholipid synthesis.	Other Metabolites
Average Diameter for HDL	Average size of HDL particles, affects cholesterol transport efficiency.	Lipoprotein Particle Concentration and Size
Phospholipids in VLDL	Phospholipids specifically in VLDL, affecting its structure and function.	Phospholipids and Lipid Composition
Cholesteryl Esters in Medium HDL	Esterified cholesterol in medium HDL particles, contributing to cholesterol transport.	Cholesterol Transport and Esterification

Figure A1 shows the distribution of improvement factors across triplet interactions. The improvement factor provides a quantitative measure of the synergy within triplets, computed as the ratio of the joint mutual information shared by two variables about the third to the aggregate of their pairwise mutual information. As shown, most improvement factors fall between 3 and 5, with a central tendency around 4, which underscores the prevalence of synergistic interactions in this dataset. This pattern implies that in lipid metabolism, higher-order interactions frequently contribute more information than the sum of individual pairwise interactions. Additionally, the extended tail of the distribution signifies that a subset of triplets possesses notably high improvement factors, emphasizing that certain interactions exhibit exceptionally high synergy. This distribution reinforces the value of exploring beyond pairwise associations to capture complex interdependencies. The top ten triplets, in terms of improvement factors, are given in Figure A2.

### Appendix A.2. Clique Algorithm Results

#### Clique Algorithm Complexity

When there is no limit on *p*, the number of cliques is imposed, the time complexity of the clique-based algorithm depends on the size of the graph and the clique size *k*. For detecting *k*-cliques, after each edge addition, the algorithm must examine the local neighbourhood of the two connected nodes to check if any new cliques have been formed. In the worst-case scenario for triangle detection (k=3), this requires checking O(n) common neighbours per edge, leading to a time complexity of $O(n^{3})$ for the entire graph, as there are $O(n^{2})$ edges in a fully connected graph. For detecting 4-cliques (k=4), the algorithm must examine combinations of pairs of neighbours, resulting in a complexity of $O(n^{4})$. Thus, in the absence of any clique limit, the algorithm has a complexity of $O(n^{k})$ for *k*-cliques, scaling with the graph’s density.

However, the aim of the clique algorithm is to quickly find a sample of strongly synergistic sets, and so a constraint is imposed to only find the top *p* sets. This significantly reduces the computational complexity. For example, if *p* = 1000, the algorithm halts after identifying 1000 cliques, regardless of the total number of possible edges in the graph. For triangle detection (k=3), each edge addition involves checking for new triangles in O(n), but since at most 1000 edges are added, the total complexity becomes O(1000·n)=O(n). For 4-cliques (k=4), each edge addition requires $O(n^{2})$ operations, leading to a total complexity of $O\left(1000\cdot n^{2}\right)=O\left(n^{2}\right)$. Therefore, with a limit of p=1000, the complexity is reduced to $O(p\cdot n^{k-2})$, making the algorithm more scalable and efficient for large graphs.

**Table A7:** Summary of PID synergy statistics for different strategies (UKB NMR data).

Strategy	Mean	Median	Std Dev	IQR
MI_ent	0.0164	0.0161	0.0039	0.0051
Inverse_MI	0.0164	0.0161	0.0039	0.0051
CH	0.0164	0.0161	0.0039	0.0051
Inverse_MI_squared	0.0164	0.0161	0.0039	0.0051
Alternating MI	0.0768	0.0541	0.0723	0.1226
MI2	0.1381	0.1334	0.0577	0.0738

**Table A8:** Proportion of PID synergy scores above ground truth percentiles for different strategies (UKB NMR data).

Strategy	Above 50th	Above 75th	Above 90th	Above 95th
MI_ent	0.00%	0.00%	0.00%	0.00%
Inverse_MI	0.00%	0.00%	0.00%	0.00%
CH	0.00%	0.00%	0.00%	0.00%
Inverse_MI_squared	0.00%	0.00%	0.00%	0.00%
Alternating MI	50.63%	39.60%	14.34%	4.62%
MI2	95.86%	75.00%	30.00%	11.08%

**Table A9:** Summary of PID synergy statistics for different strategies (YFS data).

Strategy	Mean	Median	Std Dev	IQR
MI_ent	0.0304	0.0301	0.0050	0.0068
Inverse_MI	0.0304	0.0301	0.0050	0.0068
CH	0.0304	0.0301	0.0050	0.0068
Inverse_MI_squared	0.0304	0.0301	0.0050	0.0068
Alternating MI	0.1534	0.1470	0.0785	0.0985
MI2	0.1622	0.1526	0.0736	0.0928

**Table A10:** Proportion of PID synergy scores above ground truth percentiles for different strategies (YFS data).

Strategy	Above 50th	Above 75th	Above 90th	Above 95th
MI_ent	0.14%	0.00%	0.00%	0.00%
Inverse_MI	0.15%	0.00%	0.00%	0.00%
CH	0.14%	0.00%	0.00%	0.00%
Inverse_MI_squared	0.15%	0.00%	0.00%	0.00%
Alternating MI	90.62%	87.35%	79.03%	68.86%
MI2	96.40%	92.95%	84.30%	73.76%

**Table A11:** Summary of statistics for different strategies using the O-information measure (UKB NMR data).

Strategy	Mean	Median	Std Dev	IQR
MI_ent	0.4904	0.4902	0.0252	0.0343
Inverse_MI	0.4904	0.4902	0.0252	0.0343
CH	0.4904	0.4902	0.0252	0.0343
Inverse_MI_squared	0.4904	0.4902	0.0252	0.0343
Alternating MI	−0.9126	−1.3427	0.8532	1.6449
MI2	−1.4117	−1.3430	0.2502	0.2890

**Table A12:** Proportion of scores above ground truth percentiles for different strategies using the O-information measure (UKB NMR data).

Strategy	Above 50th	Above 75th	Above 90th	Above 95th
MI_ent	99.63%	79.74%	38.96%	17.33%
Inverse_MI	99.63%	79.75%	38.96%	17.34%
CH	99.63%	79.74%	38.96%	17.33%
Inverse_MI_squared	99.63%	79.75%	38.96%	17.34%
Alternating MI	6.30%	5.13%	2.56%	1.12%
MI2	0.00%	0.00%	0.00%	0.00%

**Table A13:** Summary of statistics for different strategies using the O-information measure (YFS data).

Strategy	Mean	Median	Std Dev	IQR
MI_ent	0.2496	0.2491	0.0224	0.0300
Inverse_MI	0.2496	0.2491	0.0224	0.0300
CH	0.2496	0.2491	0.0224	0.0300
Inverse_MI_squared	0.2496	0.2491	0.0224	0.0300
Alternating MI	−0.2765	−0.2886	0.2725	0.2300
MI2	−0.3390	−0.2910	0.1886	0.2006

**Table A14:** Proportion of scores above ground truth percentiles for different strategies using the O-information measure (YFS data).

Strategy	Above 50th	Above 75th	Above 90th	Above 95th
MI_ent	59.43%	29.55%	11.73%	5.52%
Inverse_MI	59.42%	29.56%	11.73%	5.52%
CH	59.43%	29.55%	11.73%	5.52%
Inverse_MI_squared	59.42%	29.56%	11.73%	5.52%
Alternating MI	1.37%	0.37%	0.10%	0.05%
MI2	0.00%	0.00%	0.00%	0.00%

### Appendix A.3. PSO Training

Figure A3 compares the initial and final O-information scores for each particle in the PSO algorithm under a single parameter configuration. Each particle begins with an initial score (shown in blue), and progresses towards a lower final score (shown in red) as the algorithm iterates. The overall trend of decreasing scores suggests that PSO effectively guides particles to explore lower O-information regions, thereby enhancing optimization performance. Notably, the distribution of final scores below, the initial scores indicates that PSO enables particles to escape local minima, a common limitation observed in SA. This capacity for avoiding local optima is critical for accurately identifying synergistic interactions, further validating PSO as a suitable method for exploring complex interaction landscapes.

### Appendix A.4. SA Training over Epochs

Figure A4 presents the training trajectories of three distinct runs of the SA algorithm, highlighting its performance and sensitivity within the optimization landscape for identifying synergistic triplets. Panel (a) shows an instance where the SA algorithm remains trapped in a region of suboptimal solutions, with scores fluctuating around the same range without notable improvement. The lack of progress indicates the algorithm’s difficulty in escaping local minima in this particular run. Panel (b) shows the SA initially struggles to find an optimal path, leading to high score variability. However, after approximately 4000 steps, a gradual improvement emerges, enabling the algorithm to converge on a highly synergistic triplet. In panel (C), the SA algorithm rapidly identifies a highly synergistic triplet within the first few thousand steps and maintains this low score with minimal fluctuations, demonstrating a successful escape from local minima.

These trajectories underscore the SA algorithm’s varied performance, which is heavily influenced by its starting conditions. The patterns suggest that while SA can be effective in identifying synergies, it may also struggle with local optima depending on the initialization, underscoring the importance of careful tuning and perhaps alternative strategies in challenging landscapes.

**Table A15:** Summary statistics for different SA configurations on YFS_strictContToDiscrete_sturges.

Configuration	Mean	Median
Inverse_MI_geometric	−0.4867	−0.4734
MI_ent_geometric	−0.4780	−0.4648
no_weights_geometric	−0.4827	−0.4635
MI_geometric	−0.5134	−0.5081
Inverse_MI_squared_geometric	−0.4862	−0.4674
